# Experimental data demonstrating the effects of silver nanoparticles on basement membrane gene and protein expression in cultured colon, mammary and bronchial epithelia

**DOI:** 10.1016/j.dib.2019.104464

**Published:** 2019-09-07

**Authors:** Susan T. Yeyeodu, Megan E. Martin, Denise K. Reaves, Jeffrey R. Enders, Lindsey M. Costantini, Jodie M. Fleming

**Affiliations:** a Charles River Discovery Services, Morrisville, NC, USA; b Department of Biological and Biomedical Sciences, North Carolina Central University, Durham, NC, USA; c Molecular Education, Technology and Research Innovation Center, North Carolina State University, Raleigh, NC, USA; d Center for Human Health and the Environment, North Carolina State University, Raleigh, NC, USA; f Lineberger Cancer Center, University of North Carolina at Chapel Hill, Chapel Hill, NC, USA

**Keywords:** Silver nanoparticles, Epithelial cells, Basement membrane, Extracellular matrix, TGF-Beta, Intracellular signalling pathway analysis

## Abstract

This data article is related to the research article entitled “Silver nanoparticles alter epithelial basement membrane integrity, cell adhesion molecule expression and TGF-beta secretion”, available in the journal *Nanomedicine: Nanotechnology, Biology, and Medicine* [1]. This Data in Brief consists of data that describe changes in the expression of basement membrane (BM)-associated genes and proteins in three non-transformed epithelial cell lines following acute (6 h) and chronic (24 h plus 7-day chase) exposure to silver nanoparticles (AgNPs). Human BEAS2B (lung), MCF10AI (breast), and CCD-18Co (colon) cultured epithelia were analyzed for protein expression by LC-MS/MS and for gene expression by pathway-focused QRT-PCR arrays of 168 focal adhesion, integrin, and extracellular matrix (ECM) genes known to be localized to the plasma membrane, the BM/ECM, or secreted into the extracellular space. Ingenuity pathway analysis (IPA) of combined gene and protein expression datasets was then used to predict canonical pathways affected by AgNP exposure.

**Table:** Specifications Table

Subject area	*Molecular Biology*
More specific subject area	*Extracellular Matrices in Cultured Epithelia*
Type of data	*Tables (Excel files), Figures (Power Point)*
How data was acquired	*LC-MS/MS, QRT-PCR, IPA*
Data format	*Raw and Analyzed*
Experimental factors	*Cultured epithelial cells were treated with AgNPs under acute (6 h) and chronic (8 d) conditions and compared with controls*
Experimental features	*Protein expression was evaluated using LC-MS/MS; gene expression was evaluated using QRT-PCR; key regulatory pathways were predicted with IPA.*
Data source location	*Durham, NC (USA)*
Data accessibility	*Publicly available from this article.*
Related research article	M. Martin, D. Reaves, B. Jeffcoat, J. Enders, L. Costantini, S. Yeyeodu, D. Botta, T. Kavanagh, J. Fleming, Silver nanoparticles alter epithelial basement membrane integrity, cell adhesion molecule expression, and TGF-β1 secretion, Nanomedicine. Jul 24:102070. (2019) https://doi.org/10.1016/j.nano.2019.102070[1].

**Table:** 

**Value of the data** • The majority of data describing *in vitro* silver nanoparticle (AgNP) toxicity has been generated from transformed (cancer) cell lines. In contrast, the data provided in this article characterizes AgNP-induced changes in the protein and gene expression of non-transformed (“normal”) epithelial cell lines derived from lung, colon and breast tissue. Thus, these data provide baselines for comparison in future experiments that explore the pathophysiology of diseases induced by AgNP toxicity. • More broadly, the control/baseline datasets of gene and protein expression from normal lung, colon and breast cell lines available in this article can be used for comparison a) with gene and protein expression in normal or diseased lung, colon and breast tissue *in vivo*, and b) with the same or similar cell lines tested *in vitro* and *in vivo* that have been altered by disease, genetic engineering or exposure to pathogens, therapeutic agents or toxins. • The datasets available in this article identify panels of genes and proteins affected by AgNPs which embed in the extracellular matrix (ECM) and basement membrane (BM). These data have the potential to inform experiments to determine the outside-in/extracellular signaling effects of AgNP exposure on epithelial cells that support AgNP-stimulated intracellular signaling events and overall organ function. • Most published datasets on molecular changes in the ECM include either proteomic or transcriptomic data, but not both, from a variety of species, tissue sources and/or methods of sample preparation. Data provided here include the expression of both proteins and genes associated with the ECM. Comparison of protein and gene expression data can be used to reveal post-transcriptional processes such as those that regulate ECM composition during tissue repair in murine lung [2]. • These *in vitro* data provide a starting point for future, more clinically relevant *in vivo* experiments that can address the complex interactions between the ECM and diverse cell types in surrounding tissues [c.f. Ref [3]].

## 1 Data

**Fig. 1:**
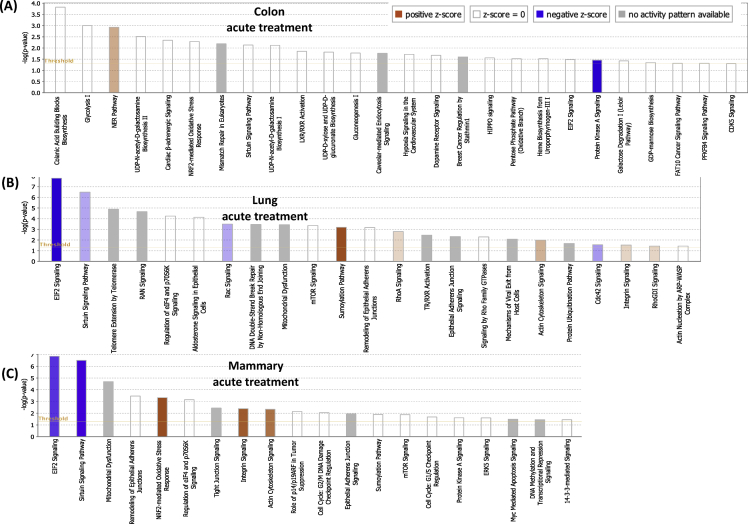
Functional analysis of proteins altered by acute AgNP exposure. Colon, lung, and mammary cell models (CCD-18Co, BEAS2B, and MCF10AI, respectively) were exposed to AgNP for 6 h and changes in protein abundance was detected by LC MS/MS. Top canonical pathways were identified and generated via IPA. Histograms show the top significant canonical pathways with each respective –log (*p*-value) on horizontal axis during AgNP exposure. Threshold *z*-score for *p-*value is indicated with a horizontal orange line.

**Fig. 2:**
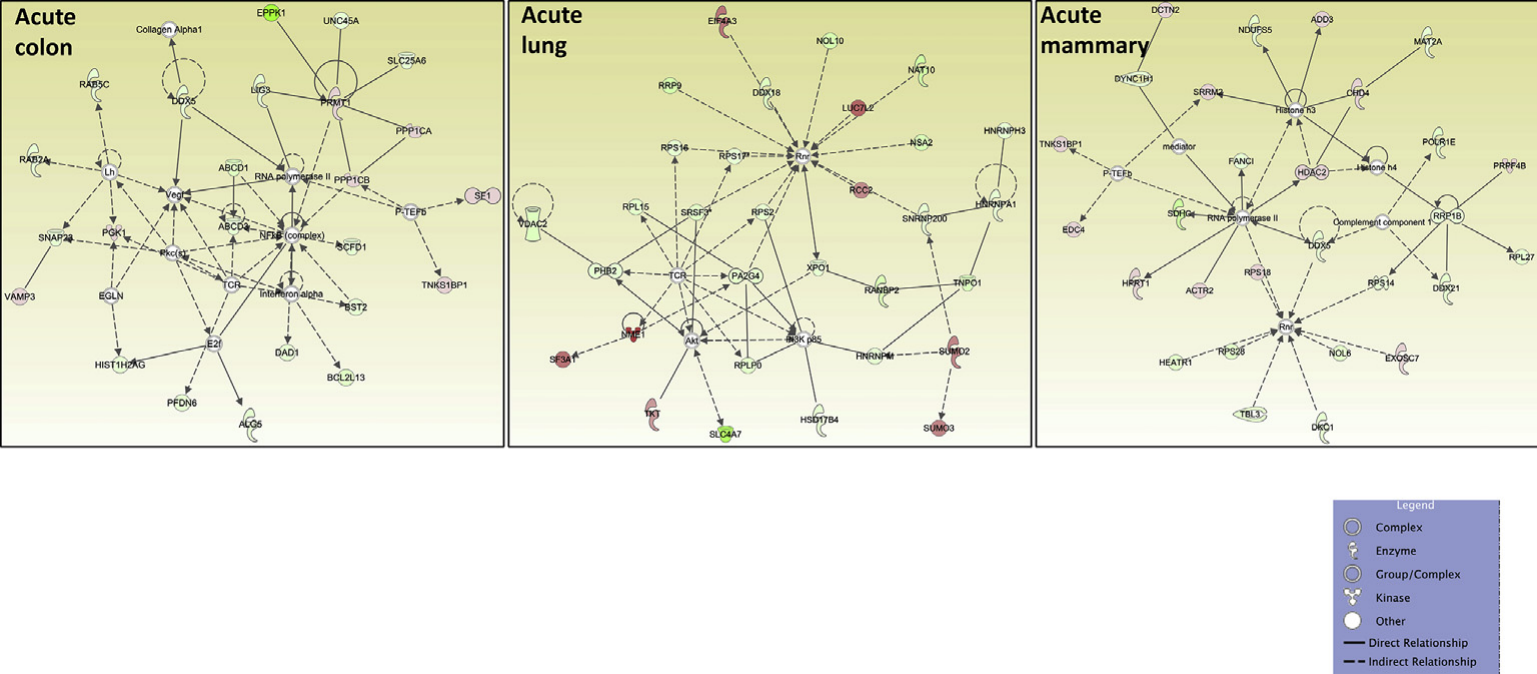
Functional analysis of proteins altered by acute AgNP exposure. Colon, lung, and mammary cell models (CCD-18Co, BEAS2B, and MCF10AI, respectively) were exposed to AgNP for 6 h and changes in protein abundance were detected by LC MS/MS. Primary causal signaling networks engaged during AgNP exposure as predicted by IPA. Lines and arrows between nodes represent direct (solid lines) and indirect (dashed lines) interactions between proteins. Red and green indicate up or down-regulation, respectively, and intensity of color indicates degree of regulation.

**Fig. 3:**
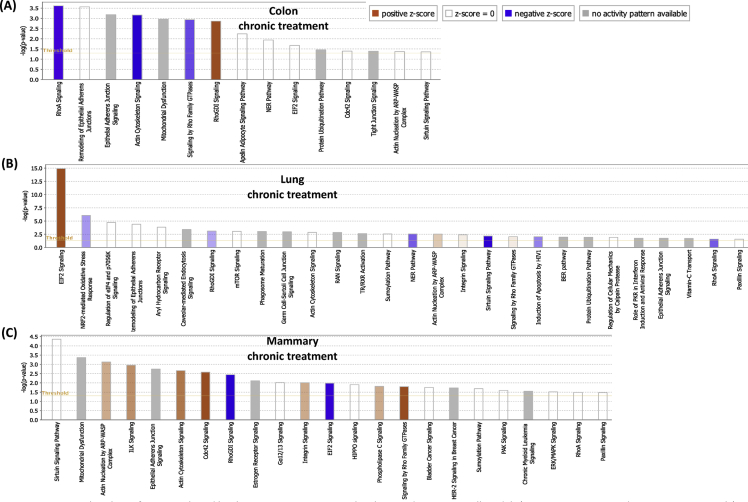
Functional analysis of proteins altered by chronic AgNP exposure. Colon, lung, and mammary cell models (CCD-18Co, BEAS2B, and MCF10AI, respectively) were exposed to AgNP for 8 days and changes in protein abundance was detected by LC MS/MS. Top canonical pathways were identified and generated via IPA. Histograms show the top significant canonical pathways with each respective –log (*p*-value) on horizontal axis during AgNP exposure. Threshold *z*-score for *p-*value is indicated with a horizontal orange line.

**Fig. 4:**
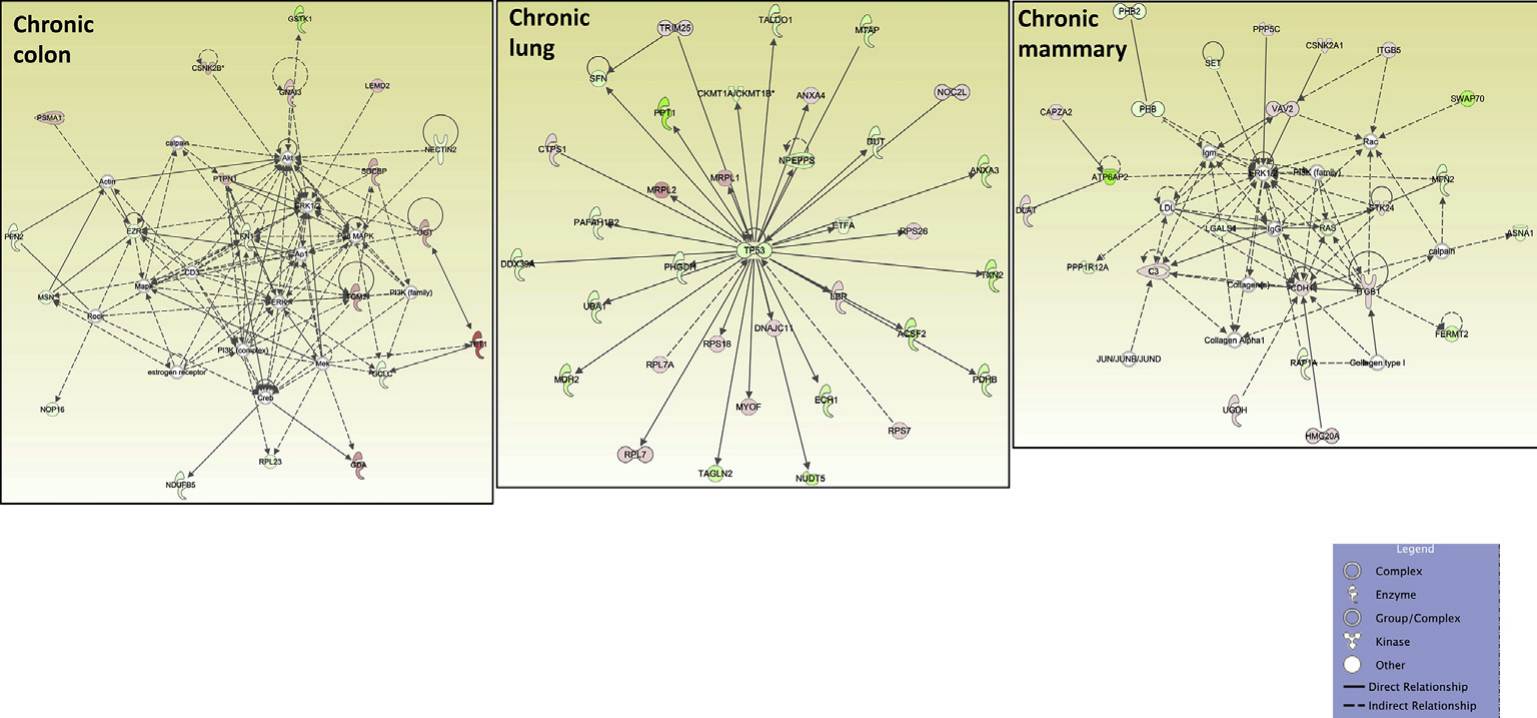
Functional analysis of proteins altered by chronic AgNP exposure. Colon, lung, and mammary cell models (CCD-18Co, BEAS2B, and MCF10AI, respectively) were exposed to AgNP for 8 days and changes in protein abundance were detected by LC MS/MS. Primary causal signaling networks engaged during AgNP exposure as predicted by IPA. Lines and arrows between nodes represent direct (solid lines) and indirect (dashed lines) interactions between proteins. Red and green indicate up or down-regulation, respectively, and intensity of color indicates degree of regulation.

**Fig. 5:**
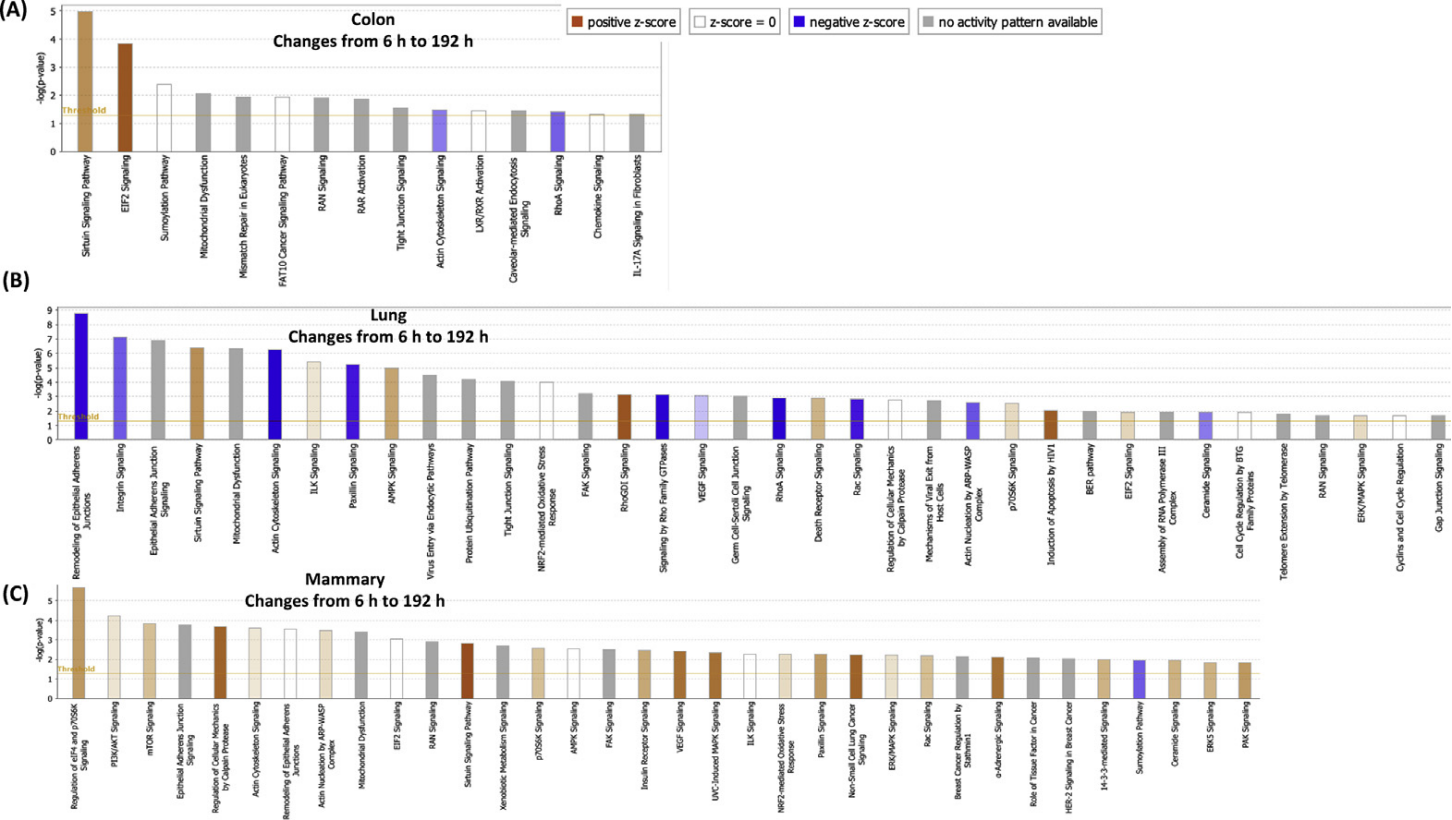
Functional analysis of proteins altered by AgNP exposure over time. Colon, lung, and mammary cell models (CCD-18Co, BEAS2B, and MCF10AI, respectively) were exposed to AgNP for 6 h and 8 days, and changes in protein abundance over time were detected by LC MS/MS. Top canonical pathways were identified and generated via IPA. Histograms show the top significant canonical pathways with each respective –log (*p*-value) on horizontal axis during AgNP exposure. Threshold *z*-score for *p-*value is indicated with a horizontal orange line.

**Fig. 6:**
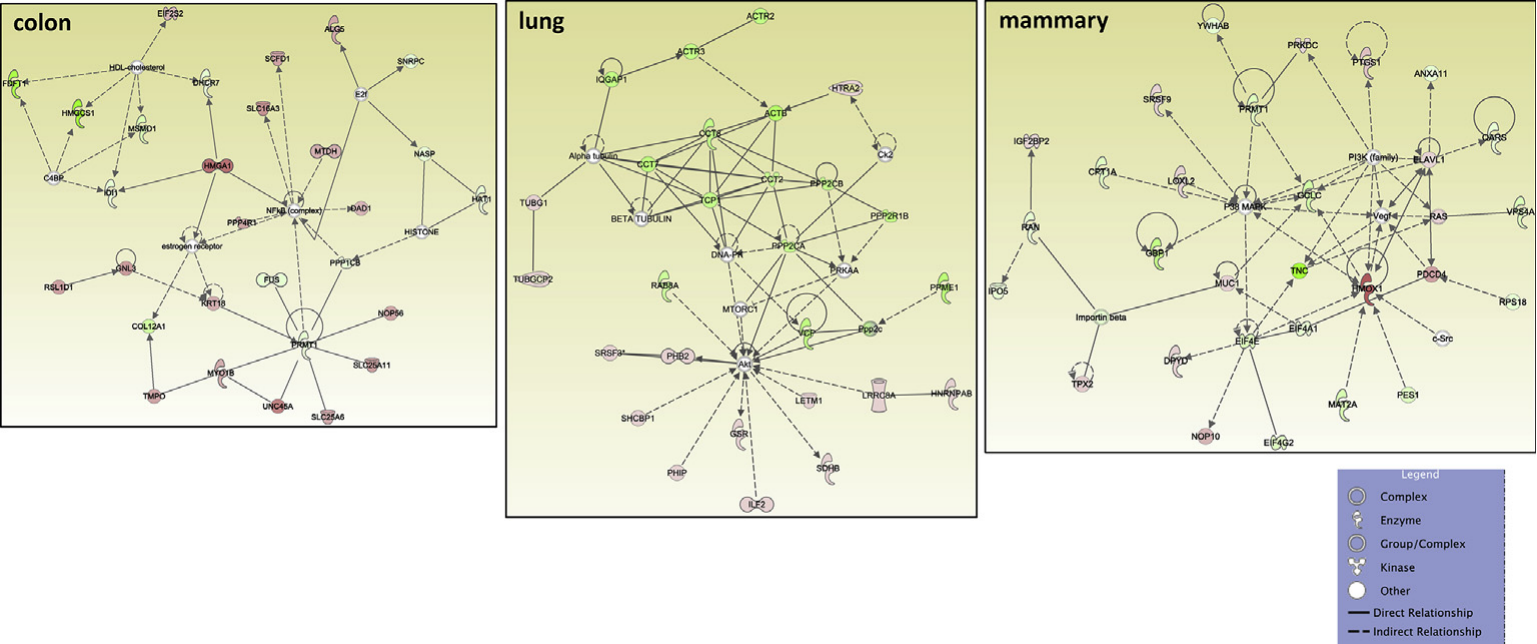
Functional analysis of proteins altered by AgNP exposure over time. Colon, lung, and mammary cell models (CCD-18Co, BEAS2B, and MCF10AI, respectively) were exposed to AgNP for 6 h and 8 days and changes in protein abundance was detected by LC MS/MS. Primary causal signaling networks engaged during AgNP exposure as predicted by IPA. Lines and arrows between nodes represent direct (solid lines) and indirect (dashed lines) interactions between proteins. Red and green indicate up or down-regulation, respectively, and intensity of color indicates degree of regulation.

**Fig. 7:**
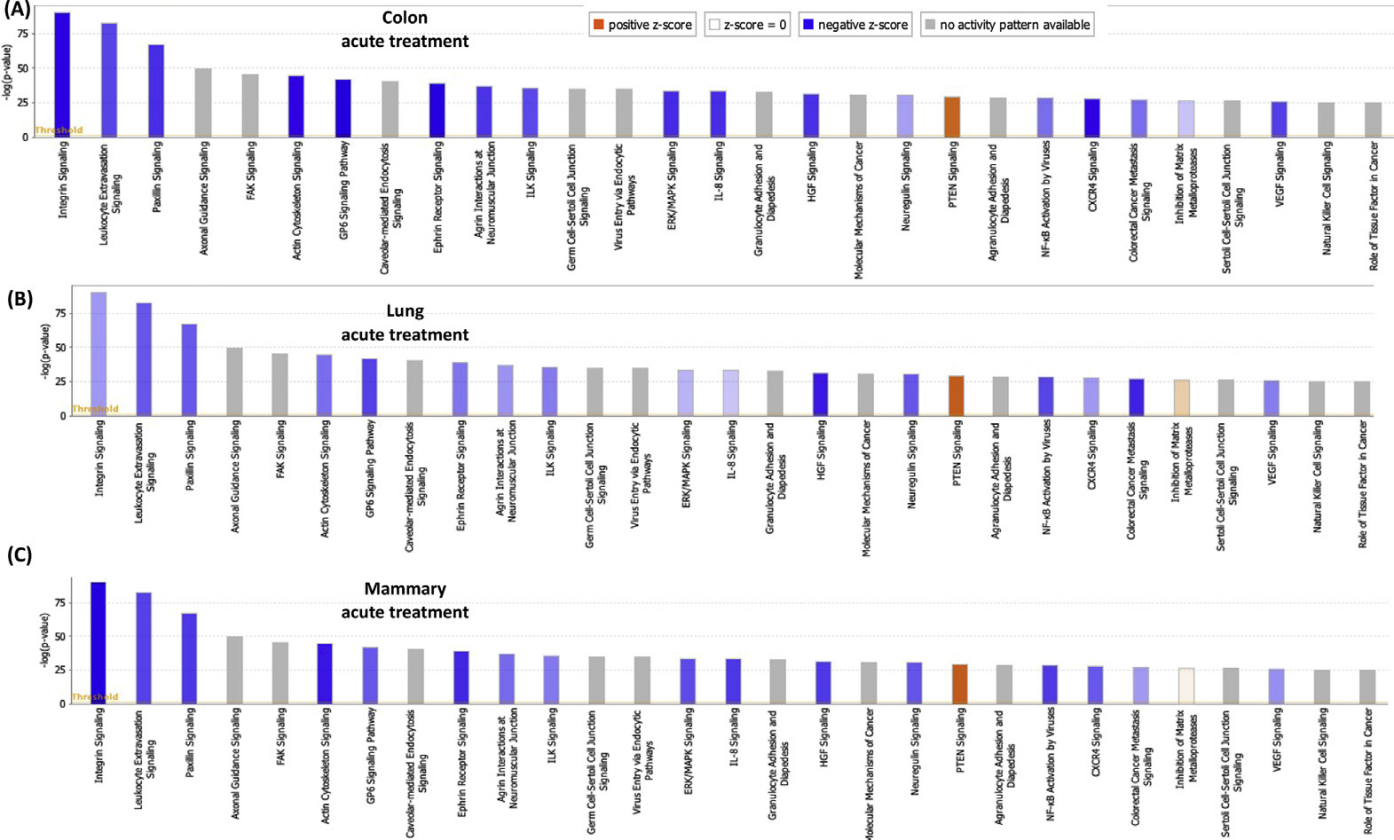

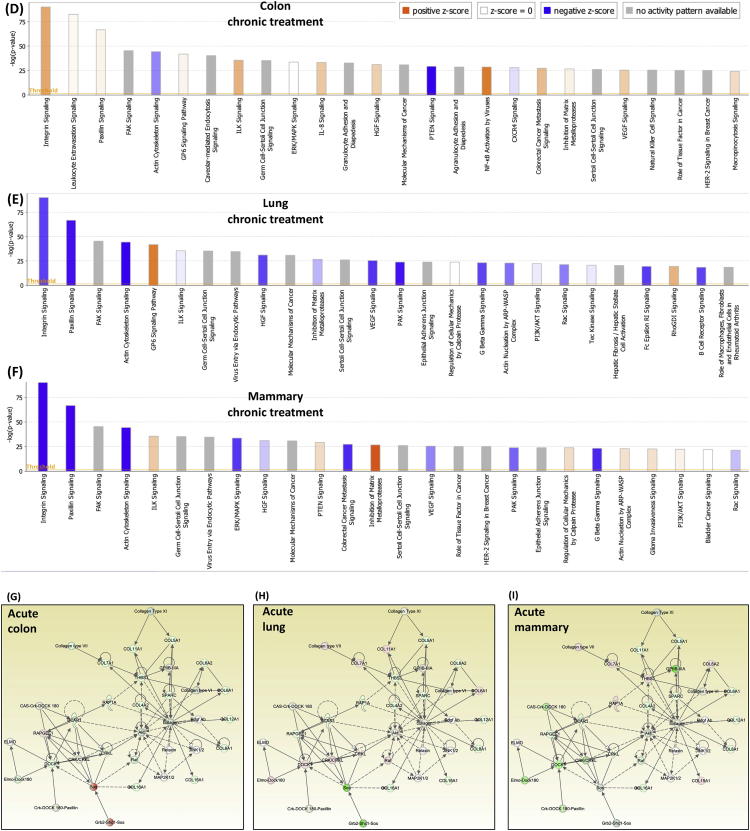

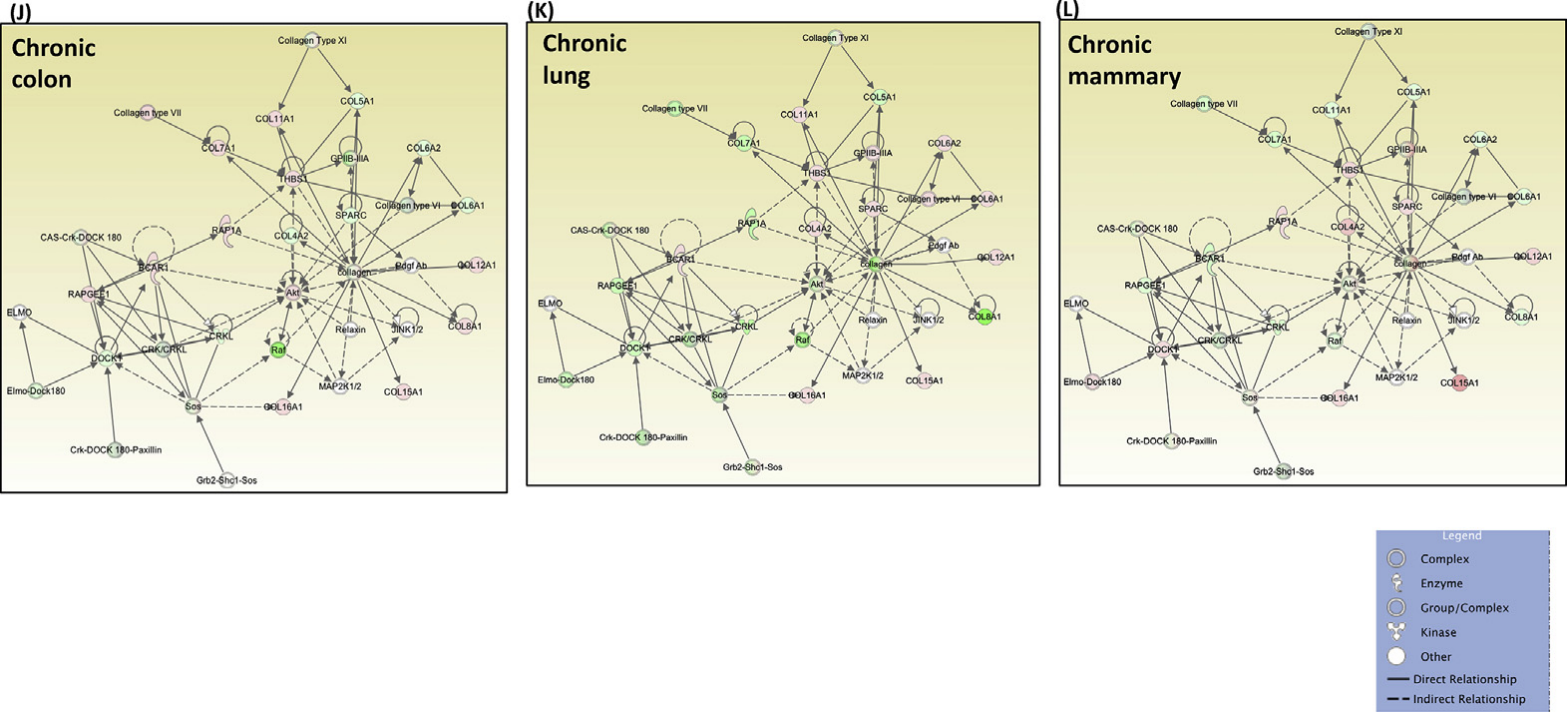
Analysis of extracellular matrix, integrin, and focal adhesion genes altered by AgNP exposure. Colon, lung, and mammary cell models (CCD-18Co, BEAS2B, and MCF10AI, respectively) were exposed to AgNP and changes in gene expression identified by pathway-focused QPCR arrays. Functional analyses of AgNP-induced changes were generated via IPA. Histograms show the top significant canonical pathways with each respective –log (*p*-value) on horizontal axis during acute (A–C) and chronic (D–F) AgNP exposure. Threshold *z*-score for *p-*value is indicated with a horizontal orange line. (C, D) Primary causal signaling networks engaged during acute (G–I) and chronic (J–L) AgNP exposure as predicted by IPA. Lines and arrows between nodes represent direct (solid lines) and indirect (dashed lines) interactions between proteins. Red and green indicate up or down-regulation, respectively, and intensity of color indicates degree of regulation.

Table 1, Table 2 summarize changes in protein expression induced by acute and chronic AgNP treatment, respectively, of BEAS2B (lung), MCF10AI (breast), and CCD-18Co (colon) cultured epithelia as determined by LC-MS/MS data. Table 3 summarizes changes in protein expression between 6 hours and 8 days in the three epithelial cell lines based on data in Table 1, Table 2. Table 4 provides QPCR array data of acute and chronic AgNP-induced changes in basement membrane-associated genes in BEAS2B (lung), MCF10AI (breast), and CCD-18Co (colon) cultured epithelia. Table 5, Table 6, Table 7 summarize IPA analysis derived from LC-MS/MS protein data of AgNP-treated colon, lung and mammary epithelia, respectively. Fig. 1, Fig. 2 illustrate the top canonical pathways and primary causal networks, respectively, associated with acute AgNP exposure in the three epithelial cell lines based on LC-MS/MS analysis data. Fig. 3, Fig. 4 display top canonical pathways and primary causal networks, respectively, associated with chronic AgNP exposure in the three epithelial cell lines based on LC-MS/MS analysis data. Fig. 5, Fig. 6 display top canonical pathways and primary causal networks, respectively, associated with changes between acute and chronic AgNP exposure in the three epithelial cell lines based on LC-MS/MS analysis data. Fig. 7A–F display top canonical pathways based on QPCR pathway-directed microarray data from the three epithelial cell lines exposed to acute (A-C) and chronic (D-F) AgNP; Fig. 7G-L display primary causal networks based on QPCR pathway-directed microarray data from the three epithelial cell lines exposed to acute (G-I) and chronic (J-L) AgNP.

**Table 1:** Molecules significantly changed by Acute AgNP treatment conditions.

Colon (CCD-18Co		Lung (BEAS2B)		Mammay (MCF10A)	
Gene ID	p-value	Gene ID	p-value	Gene ID	p-value
APOB	0.000231287625763	HNRNPA1	5.22453673611143E-06	RRP1B	0.000226525652823
ARFGEF1	0.000387774005441	HNRNPA1L2	5.22453673611143E-06	PRMT1	0.000277225136104
SLC38A2	0.000609581098547	EIF4A3	6.87382699979445E-05	PLXNA2	0.000595724408464
ANKRD28	0.000777563004275	SUMO1	0.000158217906813	TMOD3	0.000611830255566
ZC3HAV1	0.000997417455523	RPS16	0.000291671637778	DDX5	0.000828034211712
ACTBL2	0.001014735842262	PRMT1	0.000396085680618	PTGS1	0.000895634661421
ZYX	0.001346984623106	COX6C	0.00063743218428	PROM2	0.000900204274014
DAD1	0.001835370392037	HNRNPM	0.000899754279671	DIRC2	0.00108293371946
BMS1	0.002155539571948	GRHPR	0.000952778373007	SLC25A22	0.001111438313227
TET1	0.002176421762365	CLTC	0.00102559841612	SLC25A18	0.001111438313227
LGALS3BP	0.002673740447079	LDLR	0.001607596755182	SUN1	0.001116263275584
PPT1	0.003146313110455	SEPT11	0.001756524759143	UNC84A	0.001116263275584
SPC25	0.003512671124945	MT-ATP8	0.002099214535705	IFITM2	0.001234253745949
C2CD2	0.004438969494329	COPA	0.002155699935746	IFITM3	0.001234253745949
MRPL42	0.004535499380405	NOP2	0.002305914499089	PPP1R13L	0.001295201972592
TXNRD1	0.004832496990513	DDOST	0.002665187005749	AASDHPPT	0.001343264099889
MFN2	0.005164066451884	HNRNPA3	0.002707503804777	TWF2	0.001547352122358
UBQLN1	0.005204059931719	TKT	0.002762578655347	SLC25A6	0.001793098302442
PDLIM4	0.005233337791473	SLC4A7	0.00286263749303	CRNKL1	0.001818746811442
DNAJA1	0.005291134458888	HCFC1	0.003006557651275	DSG2	0.002671776404765
SRSF3	0.00568059031865	SLC38A2	0.003108020748191	TBL3	0.002943163375187
CSK	0.005976254559695	COPB1	0.003192685433245	NOL6	0.003023225724231
CD63	0.006385313301993	XPO1	0.003326097538269	MYADM	0.003097377351363
BST2	0.006393047026118	UQCR10	0.003536164258916	RAB5A	0.003229958392031
HIST1H4A	0.006963299805153	ALG2	0.003595634496192	TUFM	0.003584379653529
PIN1	0.008162424584833	SQSTM1	0.003649096724747	EIF3G	0.003639818418501
ABCD1	0.008270028881683	MTAP	0.00365891849275	COX6C	0.003656540643359
SCFD1	0.008295551069184	PHB2	0.003680460873438	STAT2	0.004114370694272
CDC37	0.008449180528454	TGM2	0.003735797493511	TPR	0.004128792357932
EPB41L3	0.008779692934559	PGK1	0.003837974426636	ZYX	0.004586543137969
CSRP1	0.008812094722484	STT3A	0.003983126512355	FAM49B	0.004623576237298
VMA21	0.009123740930331	DLD	0.004567900069862	TXNRD1	0.004803846659445
PELO	0.009181647523479	MRE11A	0.004597782505694	RPL5	0.005353032557464
FMNL2	0.009462481308876	ACOT7	0.004789157736081	EDC4	0.005512620049495
FMNL3	0.009462481308876	GAR1	0.004954045883672	EHD1	0.006362860538077
EPPK1	0.010520586220155	NME1	0.00508867370817	YWHAZ	0.006423275463892
ALG5	0.010603295146752	CSTF3	0.005303608785095	PGD	0.006490026654547
ITGA7	0.010995484265858	NOL10	0.005535613804721	TSPAN4	0.006818153191625
MRPL23	0.01118857384593	F3	0.006105317297521	LASP1	0.007471541030816
ADCK3	0.012207478208278	PI4KA	0.006186743175936	SRRM2	0.007486015366276
GPI	0.012433926051881	PI4KAP1	0.006186743175936	SUMO1	0.007570862774265
MAP1B	0.012691405109492	PI4KAP2	0.006186743175936	CYC1	0.008020043280681
UBE2Z	0.013263454104745	DHX9	0.00635577849047	GMPS	0.00810507461498
RPS8	0.013557237900314	PABPC4	0.006381937339507	CLTC	0.008597932512424
TCP11L1	0.014992103510163	ILVBL	0.006457575040699	ACAA1	0.008968965519452
EXOSC10	0.01531213171489	SNW1	0.006872111198003	LDHB	0.009145481609885
PPP1CB	0.015642280711026	SNRNP200	0.007336436965766	EPHA2	0.009201711519531
PGK1	0.015655033856218	UTP18	0.007749669700717	HINT1	0.010339271132165
VAMP3	0.015694876320621	EMC7	0.00781356791533	ENY2	0.010357399487267
IRX2	0.015764863584757	TSG101	0.007865231475941	DNM2	0.010520566525831
CPOX	0.01632320868632	SLC25A6	0.00802132872281	TMEM41B	0.010604775651278
PDLIM1	0.016659982574041	C7orf55	0.008263335751838	TXNDC5	0.010799586865328
ABCD3	0.016939515674337	LUC7L2	0.008263335751838	LIMA1	0.010902445119101
MRPS7	0.017267793214114	RPS17L	0.008352298272271	COPB1	0.010915980091817
MRPL22	0.017426787627885	RPS17	0.008352298272271	SURF4	0.010923100269767
STAT3	0.017926910993197	SNTB2	0.008683761058809	CCT5	0.011068492668342
PTRHD1	0.018081185312224	NAT10	0.009348888558558	CD63	0.011523486913144
SLC25A6	0.018323846391774	DSTN	0.009564207862485	DDX21	0.01184792321053
SNTB2	0.018514708893976	MRPS17	0.009634507997786	NUP214	0.012377399450407
TM9SF1	0.018706484886245	CNIH4	0.010097579194506	PTPRF	0.012875816281539
SLC16A3	0.018817318334633	EPPK1	0.010264940677707	TXNL1	0.012965334296902
UBE2K	0.019092948606322	PRPF31	0.01031939130842	DYNC1H1	0.013720143081198
TEX264	0.019280931663299	NSA2	0.010354924910338	CMBL	0.014255026741696
PRMT1	0.019585873076313	RCC2	0.010707968093177	MRPS27	0.015717066238882
WDR43	0.019781698932739	CAND1	0.010789032771595	PITRM1	0.01588357064183
RPS14	0.020278018860415	FASN	0.010993643748442	RETSAT	0.016743935933565
GAK	0.020395432950892	LPCAT1	0.011741620035102	NCAPH	0.016917450470554
CYB5R3	0.0205562332646	XRCC6	0.011762969543044	GNG12	0.016947400202581
SUN1	0.02108714735809	CAD	0.011881280489847	HDAC2	0.017024013497087
UNC84A	0.02108714735809	VDAC1	0.012009352959895	CDC42BPB	0.017101837162619
HEATR1	0.021327832970384	SRP9	0.012045461706109	TMPO	0.01823375774635
VDAC2	0.021349972536658	LASP1	0.01221550927402	RPL7L1	0.018235398395922
TGFBR1	0.021511283554538	PHGDH	0.012465007861747	CHD4	0.018318731088722
GNB1	0.021540662118313	CCDC22	0.012659180610054	MYL6	0.018522058276328
PRPF4	0.022199684698654	ZMYM6NB	0.013021170688271	GCLC	0.018732779452971
RABGGTA	0.022675671380617	EIF3G	0.013079608020579	SF3A1	0.018876740802136
SF1	0.022808076937076	SRRM2	0.013414883973585	EIF4G2	0.018959391066718
SLK	0.022823974978211	RTN4	0.013648201733525	USP14	0.019179519025448
GPR89B	0.02329850965464	RPL15	0.013660882591582	AGO2	0.019284383323512
GPR89A	0.02329850965464	CCT2	0.014195883308151	GSPT1	0.019779462622023
GPR89C	0.02329850965464	ZYX	0.014757729780239	GSPT2	0.019779462622023
COG5	0.023879723148424	GOLPH3	0.015249596442067	HPRT1	0.01984767514602
TNKS1BP1	0.024901205286217	DNAJC9	0.015465816960935	DARS	0.019928435750908
ATAD3A	0.025112116126869	TUBGCP2	0.015959617879774	DKFZp781B11202	0.019928435750908
C7orf50	0.025607462477173	RPN2	0.016016784839929	NAPG	0.019978083669788
COX20	0.026016463019183	MBOAT7	0.016081913266331	NCLN	0.020067924342772
CADM1	0.026507049199252	DIMT1	0.016292818734234	TOMM40	0.020521701130667
RFC5	0.026737103030205	NUP85	0.01634009411703	SAR1A	0.020724286077859
TTYH3	0.026805998055966	RAB12	0.016403760411276	RSL24D1	0.021241734165922
GALE	0.02690833138777	MYBBP1A	0.016531247739701	VDAC2	0.021381418886571
TMEM2	0.026923733507169	HNRNPA0	0.016703765974718	PPID	0.021523459034648
DHX15	0.027244051598445	SLC2A1	0.017427224134666	TPM3	0.021541792499738
DDX5	0.028028957598023	SFRS3	0.017623012021422	DKFZp686J1372	0.021541792499738
S100A10	0.02808587188919	SRSF3	0.017623012021422	VPS29	0.021549980162664
AGFG1	0.028183306162743	PDLIM5	0.01778762846831	COG3	0.021806197236079
RFC3	0.028278848051549	IMPDH2	0.017819648130724	GALK1	0.02232138134806
SLC25A11	0.028645520264937	IPO4	0.01804396610513	ENDOD1	0.022387317369289
AKAP2	0.030146185334183	PHIP	0.018205473701429	BET1L	0.022679788718574
PPP1CA	0.030262879694153	AHCY	0.018240872252383	CCT7	0.022730741149432
SPG7	0.030281021487912	GOSR2	0.018294071314393	RPS18	0.022996177165986
RHEB	0.030554567930204	SLC27A4	0.018416766205275	ALDH9A1	0.023716574165439
PFDN6	0.031201181576692	MYO1B	0.018482824386929	CKMT1A	0.024034527148651
MGST3	0.031306640128498	MPDU1	0.018557831087075	CKMT1B	0.024034527148651
PNO1	0.031307364447942	PANX1	0.019071908576041	TTC1	0.025148665897153
VIM	0.031358414796026	SDHC	0.019075634895094	NAA10	0.025175947737159
FUS	0.031412121808725	MRPL19	0.019158670533296	KPRP	0.025213621112955
SCD	0.031512655684839	SF3A2	0.019212482122539	SDHC	0.025233851313238
CIT	0.031620521865646	TOMM22	0.019727420298637	PDCD6IP	0.025403778132636
AIP	0.032014844557122	HEBP2	0.019800274167558	RPS14	0.025456143415631
TCEA1	0.032092806465569	GFPT1	0.02097993150754	PFN2	0.025552474210762
HIST2H2AC	0.032468566441941	SLC12A4	0.02113653732755	NUCB2	0.025755370532827
HIST2H2AA3	0.032468566441941	ELAVL1	0.021286176701248	HARS	0.025832193501161
HIST1H2AJ	0.032468566441941	CIRBP	0.02193919172492	MRPS26	0.025833599339365
HIST1H2AH	0.032468566441941	SF1	0.022260062809103	TRIM56	0.025925228184471
H2AFJ	0.032468566441941	SHCBP1	0.022324174309417	MPDU1	0.026011222428955
HIST1H2AD	0.032468566441941	MAL2	0.022712755154418	CNP	0.026054846147087
HIST1H2AG	0.032468566441941	CDC42	0.023415915788278	TIPRL	0.026121499386631
PFKM	0.032564784238456	SLC25A12	0.023546046832408	ADD3	0.026180587212181
PHPT1	0.033810776336437	MFSD10	0.024395218408174	RFC4	0.026445326090428
CCT3	0.034103921816157	MT-ND4	0.024797656886567	PRPF4B	0.026598192168766
CNIH1	0.034398032590271	FNDC3B	0.024908410022408	PSMB7	0.026723742098685
SEC63	0.034448863368575	SF3A1	0.024917137134698	SEC23IP	0.027272640285834
CAV1	0.03512370499182	HMGB2	0.025133472809083	RPL3	0.027448749040366
VPS4A	0.035177748776296	EDC4	0.025430230593435	TRIOBP	0.027830659966861
NIFK	0.035208710033552	UBA1	0.025451629420939	ITPRIP	0.028062120438334
DDX56	0.035335123071924	PRPS1	0.025891302966963	SCRN1	0.028202583079995
RPN1	0.035448143087613	NUSAP1	0.026279132372623	SAMD9	0.029007073507971
VAPA	0.035860077864977	VIM	0.026432420058083	DSP	0.029412852005501
RPLP1	0.036016088054509	DYNC1H1	0.026651675925698	SLC22A18	0.029536101317406
PPL	0.036078215514732	ANLN	0.026877792966651	TLN1	0.029858440714657
NCKAP1	0.036107690459537	OPA1	0.027187790423726	UBE2L3	0.029888411412698
COL12A1	0.036282319391976	MPZL1	0.02742009257935	CSRP1	0.0306249864334
LANCL1	0.036307143134736	IQGAP3	0.027549630223456	DCTN2	0.030790565236411
PPIE	0.036634493505003	NCKAP1	0.027559381462937	VDAC3	0.031492367797067
PPP1R14B	0.037008549700082	TUBB	0.028009752300089	ATP6V0D1	0.031916655797513
LIMK2	0.037008549700082	ITPR3	0.028117176896396	HEATR1	0.032619213256822
LCLAT1	0.038061507275881	TNPO1	0.028437158410456	GTPBP1	0.032674060269372
MRPL15	0.038169122052756	CD2AP	0.029060545751431	TMED3	0.033181681867598
UGDH	0.038779239507662	CENPV	0.029091933038999	YBX3	0.03331498183205
HYDIN	0.039075449883277	VDAC2	0.029179665586678	ANP32E	0.033397321701816
TMEM189	0.039747329423226	HNRNPA2B1	0.029220015831537	HMGN1	0.03356708416162
TM9SF4	0.040673054254152	RPS2	0.029461736174923	YWHAE	0.034163998120851
FXR2	0.040829706737347	EIF4G2	0.029974765886337	MAFF	0.035064083498786
PMPCB	0.040883397384934	ETFB	0.030695030868564	RAB2A	0.035171296100089
PGD	0.040912225263318	RPS15	0.030781461470498	TIA1	0.035392299549179
RAB2A	0.041162183143889	EIF2S2	0.030784172299042	EXOSC7	0.035595080898318
KIF5B	0.041669486435787	SAR1B	0.030838535857171	ATP6V1B2	0.035803052658837
BCL2L13	0.041771873917816	DKFZp434B2017	0.030838535857171	FUS	0.036441682386489
UNC45A	0.042124827954157	IGF2BP3	0.030872049584788	CLDN7	0.036546144240182
NXF1	0.042850480961424	ARPC3	0.031987116181073	PRKDC	0.037095090609212
RAB5C	0.042869367525556	ACO1	0.032117255422848	VCP	0.037102459751318
CD151	0.043266188997899	IRP1	0.032117255422848	ERP44	0.037343624594792
TIMM50	0.043394976827042	MAT2B	0.032271888399787	CSTF2	0.037420316615045
GOLGA3	0.043537126164822	APEH	0.032555166382833	CSTF2T	0.037420316615045
FNDC3A	0.044006679086649	CLN6	0.032671687397793	OPTN	0.038187216882936
TXNL1	0.044518752124354	SUPT6H	0.032723164348351	DNAJA1	0.038268287845525
KIRREL	0.045101995992972	PTPN11	0.032817102203369	NIP7	0.038463060840025
LIG3	0.045291823434148	PRPSAP1	0.033061071171445	PPIA	0.038532699912562
SNAP23	0.045895564156962	DDX18	0.033287311443996	RAB18	0.039059924662796
NOSIP	0.046113165902934	MRPL40	0.033676079849436	JUP	0.03933743261992
SAA1	0.046848901378812	RANBP2	0.03398204657942	RPL27	0.039392245139739
SAA2	0.046848901378812	MRPS9	0.034114137013208	ACTR2	0.039458476129526
NQO1	0.047747859995291	TMEM41B	0.034202794326918	NDUFS5	0.039544504706276
TAGLN2	0.047783974601158	DPY19L1	0.034611265244431	COX5A	0.039545905854819
NSUN2	0.048012722570739	HNRNPH3	0.034669293216987	AIP	0.040081683170476
ATP5J2-PTCD1	0.048371395260439	MAP4	0.035380316264461	CNIH1	0.040366441374702
ATP5J2	0.048371395260439	MRPL24	0.035385575585143	FIS1	0.040554669639993
DNAJB12	0.048413435850043	HNRNPL	0.036067160368316	RPS28	0.040670126653114
ACAT2	0.048589978572823	NDUFA7	0.036220748711523	CCT2	0.041426603041589
SLC16A1	0.04925352323993	DHCR24	0.036951786318875	SPINT1	0.041439882363767
PLP2	0.049592649783622	ACTR1B	0.036974085697414	MAT2A	0.041599571891297
PTK7	0.049644732836356	SLC25A5	0.037127662642273	FANCI	0.04183417007332
EXOSC3	0.049733274331674	STXBP3	0.037526990078928	ACTN4	0.04192774647679
		PA2G4	0.037581519046135	CCT4	0.042095800079964
		DNAJB6	0.037875156106342	RPL22	0.042811884245188
		PTBP1	0.038963008743012	SENP3	0.042869486062521
		RPL7L1	0.039003739921948	TNKS1BP1	0.043130443515044
		HSD17B4	0.039260634090604	GNL3	0.043188388290949
		SRSF11	0.039785998732668	POLR1E	0.043427195420168
		SF3B14	0.039828649381785	PTPN11	0.044023633793177
		RPL5	0.040001871164344	SRRT	0.044273264416013
		DYNC1LI2	0.040022541203206	NAE1	0.044363285311374
		RPLP0	0.040147383640671	AP2M1	0.045025010332129
		RPLP0P6	0.040147383640671	TSN	0.045138793492276
		CPD	0.040186592664847	UBA3	0.045559721745492
		PPID	0.040361707033336	PSMD8	0.046565078068495
		SUMO2	0.040395738553069	HERC4	0.046660634608427
		SUMO3	0.040395738553069	OCIAD2	0.04671856763971
		SUMO4	0.040395738553069	SLC9A3R1	0.046762209368986
		DCAF13	0.04054935551503	RILPL1	0.046789770847869
		MAGOHB	0.040581033563259	UQCRFS1	0.047702345753846
		MAGOH	0.040581033563259	UQCRFS1P1	0.047702345753846
		RRP9	0.041273246174669	DKC1	0.04795464126666
		AP2A1	0.041648103686184	TBCE	0.048603097803917
		ANO10	0.042200995103737	HIST1H1B	0.048861479341825
		DLST	0.042204489965383	EIF2AK2	0.049504002314705
		MACROD1	0.042682916500242	GFM1	0.049779715297848
		IARS	0.043206099549642	AHCY	0.049792910769453
		EDF1	0.043548325934466	PRKRA	0.049839143690143
		SQRDL	0.043613512665377		
		HSPB1	0.043806623613469		
		GOLGB1	0.04382355563834		
		DHRS7B	0.04393961099647		
		PTDSS1	0.044114769743999		
		RPN1	0.044262709794297		
		ACAT1	0.044294656309863		
		MYL9	0.044658311671535		
		PAFAH1B1	0.044912251394459		
		VPS4A	0.045022611148262		
		AKR1B1	0.045111729481386		
		MYADM	0.045150497042427		
		MAN1B1	0.045162773746598		
		ENO1	0.045458068411755		
		DNAJC13	0.045478443240726		
		PLEC	0.045478628559484		
		PRKDC	0.045669263041687		
		ATAD1	0.045674530590761		
		PKM	0.045902509313299		
		KPNB1	0.046125946922567		
		SRGAP1	0.046549140051299		
		SRGAP2C	0.046549140051299		
		SRGAP2	0.046549140051299		
		ESYT1	0.046730563360088		
		NCAPH	0.046861455420858		
		SNRPC	0.046869684140825		
		CS	0.04755599026804		
		PSMD11	0.048438884346468		
		GSPT1	0.048545960403953		
		GSPT2	0.048545960403953		
		HSPD1	0.048720365410717		
		ARPC4	0.049320443585959		
		ARPC4-TTLL3	0.049320443585959		
		UBE3C	0.049377611892574		
		NPLOC4	0.049516218669462		
		PREB	0.049695906471427

**Table 2:** Molecules significantly changed by Chronic AgNP treatment conditions.

Colon (CCD-18Co)		Lung (BEAS2B)		Mammay (MCF10A)	
Gene ID	p-value	Gene ID	p-value	Gene ID	p-value
NOP16	0.000102267436579	GSTP1	7.45643995698985E-06	HYDIN	0.000816188767313
GPX1	0.000152579246674	ALDOA	0.000126669607621	WDR75	0.001129968106219
GCLC	0.001577772847639	ENO2	0.000128617761485	RAP1A	0.001146283517896
ACOT7	0.001701546468697	PDCD5	0.000231705348233	ALCAM	0.001198374611824
PPIE	0.002197984924673	RPA2	0.000239028999395	SF3B3	0.001690834284681
RPL23	0.002812948977478	UBE2V1	0.00029827135473	IGF2BP2	0.003138310869382
METAP1	0.00364559525432	PRCP	0.000496925549926	PCK2	0.003391334688521
EEF1E1	0.005028364742025	MYDGF	0.000563091741403	ALDOC	0.003704193814977
CSNK2B	0.006606345090918	NOC2L	0.000678889595585	RPS15	0.003761814029399
RPS2	0.007177169322026	PHGDH	0.00084050762021	ARPC5	0.004579138490763
UPF1	0.007804331612779	PLS3	0.000938938956854	SWAP70	0.005302059813726
TET1	0.008133468633597	HACD3	0.000986588415963	DARS2	0.006429605928229
NOP9	0.009155197498028	GDI1	0.001421557721266	HMOX1	0.006584083999339
IER3IP1	0.010264583234286	TPI1	0.001676897491934	UBE2I	0.006606767027723
DUT	0.010323661215839	HM13	0.001795054222325	CDH1	0.006641949572697
EML2	0.010348056747955	TCEA1	0.001856079700328	NUP62	0.007022285827574
HMGN1	0.010744683887679	DR1	0.002059000262156	CAPZA2	0.007052340265518
PVRL2	0.011380138867175	WASL	0.002123331494806	PYCARD	0.007112389756527
VAMP3	0.01204909230113	GSTM3	0.002158151177994	SLC35E1	0.00724612147683
NDUFB5	0.012714595059011	TBRG4	0.002172283740892	CWF19L1	0.007719032917852
FBLIM1	0.01283437873768	BLVRA	0.002258835500326	LGALS1	0.007859290736339
ATP6AP1	0.013162524809982	NIT2	0.002354704296246	MRPL40	0.009043559941229
NMNAT1	0.013248987410444	HDGF	0.002760620843881	SEC14L2	0.01112202343708
STT3B	0.014094839318089	MDH2	0.002822109996685	GEMIN4	0.011320673187743
BBOX1	0.014713087359506	RPL7A	0.002833294156389	SLC22A18	0.01133567223914
WDR26	0.01532692379904	RPL22L1	0.003059963371581	ATP6AP2	0.011649916970073
MSN	0.015697051512636	COL12A1	0.003150938670078	ERP44	0.01198952805729
EZR	0.016505993113143	TBCA	0.003284428202612	TPD52	0.013175063090262
EXOSC3	0.017112656177424	HEBP1	0.00335798962136	RPL32	0.013659249151337
SDF2	0.01746491417822	BCAT2	0.00357374823763	FERMT2	0.014273172200358
COPS2	0.017919843720826	HADHA	0.003642307549926	DLAT	0.014991339737263
ARFGAP3	0.017938217652366	ACTR3	0.003691625984744	RBM10	0.015078237143828
RPL34	0.018087930017053	ENO1	0.003726618759228	STXBP3	0.015094689912414
MSH3	0.018120841975091	SLC25A13	0.003839028443258	NDUFV2	0.015216133011573
AGRN	0.018259205965206	CNDP2	0.003887471798332	CTBP2	0.01571033734899
LAMB1	0.018394118203222	GPI	0.004088592009321	MRPS27	0.016049228324964
ETFDH	0.018413514414389	PFN1	0.004217920463606	C8orf33	0.016212682083836
SEC22B	0.020068658622312	RPL4	0.004266145237169	PHB	0.016676148233628
PSMA1	0.021530970909853	HP1BP3	0.004446105859121	ERGIC1	0.017164096487019
SDCBP	0.022379034486178	BCCIP	0.004453404363675	FNDC3B	0.01733179768575
GPD2	0.02244517685837	CLIC1	0.004491061119877	SPRYD4	0.017358748908178
GSTK1	0.022491445710759	AGTRAP	0.004625687898471	SEC63	0.017628335036449
UBE2D2; UBE2D3	0.022884210788563	RBMS2	0.004672356043434	SLC25A12	0.017663960504409
FBXO30	0.023178708974589	DIABLO	0.004900543683575	BLVRA	0.017668963917925
RPS27L	0.023257577801858	TPP1	0.00499127435589	RPL15	0.018263220710871
NPC1	0.023730183889373	NUTF2	0.004994813525281	EHD2	0.019178631343528
TMED1	0.023784086587189	CANX	0.0053394938392	PPP1R7	0.019890756990299
SEL1L	0.024431857961076	FAHD1	0.00554526112985	SNW1	0.020424152770559
GPR89B; GPR89A	0.024860017069391	OSBP	0.005582687966443	ACOT7	0.021322909477462
RNPS1	0.027039898591936	RPL17	0.005598867277088	PGAM1	0.021685513903222
LEMD2	0.027226398278768	ACTG1	0.005902721469646	IK	0.022613575231829
SUMO1	0.028478544004075	KATNAL2	0.005959784104908	SET	0.024090601594695
PFN2	0.030529395586625	APEX1	0.006195861425584	MOB1A; MOB1B	0.024632931209177
EPB41L2	0.030687328957884	HNRNPA2B1	0.006224403844595	HMG20A	0.024793347476798
CERS2	0.030760542209042	DNAJC11	0.00643541409521	PPP1R12A	0.025273448815583
NDUFA13	0.030887729364763	GDI2	0.006475868335677	RNMT	0.02549888476998
PARP4	0.030985287147997	HMOX2	0.006562776494759	FAM98A	0.02615839126873
TOR1AIP2	0.03150295320592	HEXB	0.00657735650682	YARS2	0.026372003274182
PTPN1	0.031796568770893	ALDH7A1	0.006680417144639	DLST	0.027570698608198
KIAA0020	0.032336647896505	PEBP1	0.006967037529185	SEC61A1	0.027930312444246
CDS2	0.033367800213701	MTFP1	0.007136263958499	SLC44A2	0.027989241567404
SNTB2	0.033371674760233	GFPT1	0.007224998955874	STK24	0.028223886282563
ANO10	0.033438683455801	LARP4	0.007270799245228	XPO7	0.029137392190364
SRP9	0.034304532705989	ACO1; IRP1	0.007275580887762	ITPRIP	0.029409444451346
NEB	0.035073625381054	GGH	0.007311654716582	CASK	0.029967597448605
TGM2	0.035285677361793	DUT	0.007358727957047	ACO1; IRP1	0.030153972421968
FN1	0.035678964400989	RPL13A	0.007367668247224	ITGB1	0.030154993071213
UNC45A	0.036734114544859	NUDT5	0.007619239415585	RPL3	0.030176307065026
GNAI3	0.037149135902154	GLB1	0.00770766079442	SIN3A	0.030243131245225
TFB1M	0.037334019512378	NNT	0.00783006302382	IFI16	0.031010435035415
VAMP7	0.037857137210366	CNIH4	0.007948332026921	P4HA2	0.031051204234091
RPP30	0.037922502625981	SCARB1	0.008187002846514	LIN7C	0.031465089586857
PHPT1	0.038158051644266	TALDO1	0.008200645900475	NCBP1	0.031504185726774
KPNA3	0.038531334601815	SRPRB	0.008263869532791	AKR7A2	0.031537408696946
MRPL42	0.038559733995003	SSR4	0.008266804862299	MAL2	0.031616192036917
ARPC5L	0.039688184018198	PSMB3	0.008457859926762	DDX27	0.031627414853244
ARPC1B	0.040725053810991	ACSF2	0.008724540123793	ARL8B	0.032040754384568
EMC7	0.040758316545541	TXNDC17	0.009350078186141	CNDP2	0.032618013897939
SPR	0.041036762551352	SCCPDH	0.009524082336948	CSNK2A1	0.032626600872834
BRI3BP	0.042092293615321	ANXA3	0.009580844233407	LRRC8A	0.032749601873397
UQCRQ	0.042679557753263	UBA1	0.009606655538542	ADCK4	0.032831701379408
MYCBP	0.042683637716761	GLO1	0.009621513604419	TMEM205	0.033165352792111
GDA	0.04295574609257	TGM3	0.009640267569003	CNIH1	0.034332807244484
SAFB	0.043062927875486	PTCD3	0.009710074486304	ETHE1	0.034871305459349
OGT	0.045182640527177	ITGA2	0.010014514494091	VAV2	0.035450630842936
ATP2B4	0.045286029081919	RPL29	0.010050987918552	MFN2	0.036604484629507
SEC23A	0.045397496085182	PRDX5	0.010123686033618	SMARCE1	0.037191466868621
TUBA4A	0.045717549560607	PTMA	0.010144442407195	DDX52	0.037249302710433
CYB5A	0.046202953031268	RAN	0.010230053798161	CYC1	0.038423669924853
SCAMP2	0.046835574077924	ECH1	0.010243395706381	C4orf27	0.038462544731574
NUP62	0.047117250609744	PARK7	0.010296287817891	VDAC1	0.038873925768868
STX17	0.047692253910341	CS	0.010442790042035	LMF2	0.038938752428805
TOMM70A	0.048248790705325	ETFB	0.010474937477572	NDUFS1	0.039069794233402
UBE2I	0.048459491964116	TRIM25	0.010566382108713	SPCS1	0.040198125041144
CLIP1	0.048936307199615	HIBADH	0.010580578640405	DDX1	0.040368668280252
LRRFIP1	0.049177168904081	SRSF9	0.01075400769739	FIP1L1	0.040472111913078
GOLGB1	0.049889433421408	RPS26	0.010854641326663	TMED4	0.040499151069757
		PFKP	0.010924255807384	BET1	0.040547151528895
		RPL6	0.011035564185991	RBM14	0.040789398734788
		KPNA4	0.011035966452746	RPS19BP1	0.041010820137085
		RPS8	0.011037766536237	SDHB	0.041068347067555
		EMD	0.011235044257754	TMEM147	0.041505677162942
		NME1	0.011443976251276	DDB1	0.042437267755066
		MRPL13	0.01155874592855	BAZ1B	0.042689860135034
		RAC2	0.011898150839041	EXOSC6	0.042963657988297
		DLAT	0.012381156352691	ZC3H15	0.043030716387912
		SRSF1	0.012491961236189	MRPL38	0.04352164469537
		RPL27	0.012812595090028	TRIP13	0.043776323959272
		PCMT1	0.012944682584357	ASNA1	0.044335927825863
		HSPB1	0.013101233652799	TSPO	0.044654389849355
		ANP32E	0.013359893401875	UGDH	0.04470230735611
		QARS	0.01341303073706	PPP5C	0.044861830134044
		FASN	0.013617998216036	MYL6	0.045677428882186
		CD82	0.013627123917691	PDLIM7	0.045934551610414
		RPL3	0.013750327944981	C3	0.04652278437984
		PSIP1	0.014010182827145	HARS	0.047086082489919
		LEPREL1	0.014183108113014	OGFOD1	0.04740847939116
		PHB	0.014259234000647	ITGB5	0.047414684143427
		LMAN1	0.014318142154276	PHB2	0.04745158976481
		CTSL	0.014359913906275	ARID1B	0.047904475240394
		MIF	0.014391813288184	NUDCD2	0.048334553467461
		NDUFAF2	0.014464278489264	POR	0.048590368609121
		BCAP31	0.014465942074504	UBAP2L	0.049707916926706
		HINT2	0.014627253424083		
		STIP1	0.014774840127175		
		RRS1	0.015066179313672		
		HSD17B12	0.015253959570474		
		CYCS	0.015298213471277		
		STMN2	0.015354623735198		
		CLPB	0.015721285848007		
		COA3	0.015901890669628		
		DTYMK	0.015920128066351		
		RPL14	0.015965234370569		
		AK2	0.01603672968963		
		LRBA	0.016042456528674		
		RPS13	0.016369253978614		
		NASP	0.01637507557196		
		IMP3	0.016383334287533		
		ITGA5	0.016632469745852		
		UBE2M	0.016753870099399		
		GYS1	0.016921619971655		
		RNPEP	0.017179503180866		
		FKBP8	0.01726038798928		
		UBE2N; UBE2NL	0.017299740359642		
		RPS18	0.0173924391667		
		PCNA	0.017498961663866		
		PAFAH1B2	0.017645052422399		
		RPS3	0.018032729224429		
		LAMB3	0.018154146579258		
		UBE2I	0.018161955843411		
		PPIH	0.018196816215514		
		MLLT4	0.018247001000656		
		SOD1	0.018612615105467		
		CHCHD3	0.018644297733898		
		TUBB3	0.018807414763783		
		SLC16A3	0.019033444053568		
		ARL3	0.019536711267107		
		SRSF6	0.019647282809113		
		PDHB	0.019798806030319		
		CIRH1A	0.019842393135787		
		OASL	0.019859061761995		
		ACSL3	0.020237339213001		
		STOML2	0.020483679909488		
		CLTC	0.020693951401821		
		SMNDC1	0.020718039952823		
		LBR	0.020774356704003		
		RPL7	0.020889510422727		
		NPEPPS	0.020893976821206		
		PTGES3	0.02093107189285		
		RDH11	0.020969398043848		
		CTSD	0.020994910274986		
		SLC35F6; C2orf18	0.021080045656798		
		PEA15	0.021172190221945		
		NUDCD2	0.021175350280847		
		HMOX1	0.021348623708601		
		GSTO1	0.02180736635184		
		TNKS1BP1	0.02180739580043		
		PRDX2	0.021822394220515		
		TMEM230	0.021865082580508		
		SFN	0.021930723293761		
		RPS9	0.021994141047509		
		SFXN1	0.022434755987925		
		TP53	0.02243964826995		
		GLOD4	0.022454168156741		
		CLIC4	0.022876768089019		
		RPS4X	0.02294406975963		
		IDH3A	0.023170788889634		
		TXN2	0.023422139415138		
		RANBP1	0.023596098515313		
		ANXA4	0.023657669538504		
		ECHS1	0.023665764895205		
		KDSR	0.024162102451466		
		EZR	0.024448111842932		
		RPL35	0.024472390973923		
		NQO1	0.024610661666055		
		SSR3	0.024645181015016		
		RPL8	0.024871672974189		
		NSF	0.02541040191879		
		PUF60	0.026144164026018		
		DDAH2	0.026199371702795		
		MAPRE1	0.026364088245755		
		COL17A1	0.026647641160506		
		C7orf50	0.026817718058601		
		BSG	0.026903086460419		
		LRRC1	0.027221426515277		
		ARHGDIA	0.02737338438047		
		ETFA	0.027607834567285		
		SERPINB3; SERPINB4	0.027759034097406		
		MTPN	0.027892153494141		
		DHCR7	0.028214097200114		
		MACROD1	0.028435002356811		
		IDH3B	0.028578067958511		
		GCN1L1	0.028791187902989		
		RPS7	0.028795839381372		
		COX20	0.028943226138703		
		EIF2AK2	0.029121979799936		
		TAGLN2	0.029182400239015		
		DARS	0.029208853138582		
		NAMPT	0.02934103459428		
		CKAP4	0.029350177121742		
		CFL2	0.029814548089492		
		CALR	0.029828201431842		
		MBOAT7	0.029922447134157		
		VCL	0.030135234063699		
		COPS3	0.030237003948058		
		TECR	0.030259523093885		
		RAB5B	0.030945805476466		
		PPAT	0.031350837794656		
		MYOF	0.031650866367988		
		ELAVL1	0.031825212710529		
		PPT1	0.031938331760119		
		RPL5	0.032082426815355		
		AKR1A1	0.032318025627655		
		PKP3	0.032358086286257		
		ACOX1	0.032806969764807		
		CCRL2	0.032961364799662		
		FUS	0.0332399255545		
		TMCO1	0.033241569937229		
		APOA1BP	0.034104089255631		
		MOB1A; MOB1B	0.034421159540398		
		PRC1	0.034461460172348		
		LETM1	0.034589987285492		
		AIMP1	0.035315463826148		
		MRPL1	0.035408240298784		
		ELAC2	0.035636084242757		
		SKIV2L	0.035649285140241		
		RPL31	0.035948001771965		
		UBE3A	0.035967989168347		
		ACADVL	0.035992525562705		
		SF3A2	0.036048954876786		
		ATP2A2	0.036466380659332		
		CTPS1	0.03714262636898		
		CLPTM1L	0.037312665489251		
		NNMT	0.037600543126092		
		VDAC1	0.038548770287204		
		TOP1	0.038807106980567		
		ROMO1	0.038935314839272		
		STK10	0.039084553169413		
		APOL2	0.039480404371444		
		CKMT1A; CKMT1B	0.039852857975913		
		AKR7A2; AKR7L	0.040008240097819		
		ITGA6	0.040052649049214		
		SCRN1	0.04006006154094		
		PABPC1; PABPC3	0.040404684567024		
		CRYZ	0.040440887333519		
		SLC2A1	0.040627991197916		
		IARS	0.040677613011327		
		STMN1	0.040919612076939		
		CD151	0.041130255280449		
		DDX52	0.041193945322288		
		FSCN1	0.041247681191657		
		MTAP	0.041734409025387		
		PTGR1	0.041984390644271		
		PLP2	0.042344164521116		
		NIFK	0.042488180020558		
		HYOU1	0.042818692032959		
		WDR36	0.043062702013525		
		ERP29	0.043169739840048		
		AKAP2	0.043274338130695		
		EWSR1	0.04364686388609		
		IDH2	0.043669739530499		
		DDX39A	0.04448000475581		
		IFIT3	0.04451113083264		
		MITOL	0.044831227430505		
		PABPN1	0.045415748105986		
		DDX39B; hCG_2005638	0.045936894486145		
		MDH1	0.046186040623421		
		SLK	0.04634823334454		
		ANXA11	0.046931526835144		
		DIMT1	0.046993980399505		
		SF3A3	0.047190951854311		
		LARS	0.047502461149355		
		ZC3HAV1	0.047699297295937		
		PTRF	0.047823730748009		
		MX2	0.04860036239019		
		MRPL2	0.048817239934627		
		LDHB	0.04901515070203		
		MTX2	0.049305989013682

**Table 3:** Molecules significantly changed by AgNP over time (6h–8 D).

Colon (CCD-18Co)		Lung (BEAS2B)		Mammay (MCF10A)	
Gene ID	p-value	Gene ID	p-value	Gene ID	p-value
BMS1	8.90219940492576E-05	PHGDH	1.8053442924279E-05	CRTAP	1.38824578153055E-05
EARS2	0.000580512548097	FANCI	0.000145735560319	CRNKL1	0.000124692932315
PFN2	0.00085416472985	CA9	0.000218124378047	CDV3	0.00014104323199
TMEM43	0.000958499854163	PPIB	0.000417218290915	MRPS6	0.000320024565658
PRMT1	0.00109574608526	DIMT1	0.000424690095643	PTPRF	0.000429600146451
SEMA4B	0.001555472521316	HNRNPA3	0.000428502138495	TUFM	0.000740275207812
ATP2B4	0.001698930474385	KPNA2	0.000502793916883	PROM2	0.001133964467288
NDUFAF2	0.001700512654477	AGPS	0.000642394237796	ADCK4	0.001276347656479
MSMO1	0.001735738155222	HNRNPA1; HNRNPA1L2	0.000651481635001	VKORC1; PRSS53	0.001397488248341
GLG1	0.001904481206718	PHIP	0.000660206476014	EEF2	0.001510762439164
MFN2	0.001937382622799	MDH2	0.000707464937396	NOP10	0.001615975047584
UQCRQ	0.002022181810998	KATNAL2	0.000754502222013	PTGS1	0.001742414963999
PAICS	0.002062992348856	SSB	0.000852598974295	PRMT1	0.0017840349926
SLC16A3	0.002175446171388	GAR1	0.000879257749421	EEF1D	0.001897354999761
DSTN	0.002284460834409	PDCD4	0.000929801918333	CA12	0.001906439027995
SEC61A1	0.002312671099911	MECP2	0.000936170604018	ACADVL	0.001909492796127
RAB2A	0.002815434658227	43354	0.00101331201271	CDC37	0.002067185848874
DDRGK1	0.002839622736533	ATP5A1	0.001020283817231	ARL8B	0.002164715238048
PPA1	0.003423965354165	APEX1	0.001030859779331	PTK7	0.002920589471035
PPP1CB	0.003525865383642	ACSL1	0.001070134046221	HMOX1	0.002975925713993
ACAT2	0.003526189077429	CS	0.001205001577414	FOSB	0.003265700786809
MRPL41	0.003576656281789	DHX9	0.001235655502854	EEF1A1; EEF1A1P5	0.003328714888703
VAMP3	0.003689449187691	ACTR2	0.001287843721569	NDUFAF4	0.003341951335523
ACOT7	0.003984937595472	DNAJC8	0.001297209919875	AASDHPPT	0.003503501903208
NSUN2	0.004014433732789	IQGAP1	0.001341191265058	VRK1	0.003543184529049
PTDSS1	0.004259517739362	HMGB2	0.001413715314304	TRIM56	0.003716819878129
NASP	0.004489983733883	SEC61B	0.001519755901349	ATP6AP2	0.003863786968723
RPS9	0.004591240375858	PHC2; PHC3	0.001697507514262	DDOST	0.00389766025237
FAR1	0.004690589048227	ELAVL1	0.002066169578682	KDSR	0.004325605942177
TPD52L2	0.00471358433446	SF3A2	0.002180795977084	GRB2	0.004357270702677
GAK	0.004786717870142	TUBG1; TUBG2	0.002260204503535	SRSF4	0.00440347433841
PVRL1	0.004820788002019	NCL	0.002318901448865	PTRHD1	0.004434544932656
LDLR	0.004943632169242	PPP2CA; PPP2CB	0.002350536128954	SF3B3	0.0044986214236
FKBP8	0.005260313885939	HMGB1; HMGB1P1	0.002394337896824	RBM3	0.004503047496039
HM13	0.005451132510723	AATF	0.002396462580377	DNAJA1	0.004544591036216
SNRPB	0.005786639734548	LPCAT1	0.002403755779022	IGF2BP2	0.004574932401217
DPYSL2	0.005919292000497	NOP2	0.002757058733241	TMEM33	0.004659806381862
RPL18	0.005980138056627	SNRPA1	0.00285943477434	ADSL	0.00472196595539
PGRMC2	0.005988866495383	MCM3	0.002905159287196	LRRC1	0.00472351451803
DUT	0.006075622651652	PDCD6IP	0.002953262365103	FAM162A	0.005025312275628
MRPL15	0.006185025601255	ITGB4	0.003065248157419	PRKDC	0.005353105160119
CPT2	0.006401739170173	EIF5A; EIF5AL1	0.003100883897571	STEAP3	0.005450725756852
AFG3L2	0.006795252893284	ILF2	0.003106168481721	LARP4	0.005637114159459
SLC39A14	0.006801725054794	CLTC	0.003228332897914	MOB1A; MOB1B	0.005887523661818
SDF2L1	0.006829834464515	NDUFS4	0.003422145347307	MAT2A	0.006149498474513
HADHA	0.006923780290653	DPY19L1	0.003427706260351	CCT2	0.006361637078673
PTGES	0.007167765644743	DSC2	0.003690410005985	CASK	0.006576503432456
MRPL22	0.007244888413221	ASAP1	0.003707216176263	TET2	0.006592349501587
NAMPT; NAMPTL	0.007407158263087	ARPC4; ARPC4-TTLL3	0.004104736983589	PHACTR1	0.006721957485993
STAT3	0.007781233826464	DYNC1LI2	0.004224244785702	MYADM	0.006900752150609
DKC1	0.008252890211008	HSD17B4	0.00428490715789	EIF3G	0.006984531211746
TMPO	0.008608706360001	EIF2B4	0.004288019362093	TOR1AIP1	0.007039465915218
PRDX1	0.00864601722588	PSMB3	0.004364944519813	LRP1	0.007268283795628
MAN1B1	0.008992235234772	ACTG1	0.004437513772166	ERVMER34-1	0.00781532697896
CYB5R1	0.009002989284376	IGF2BP2	0.004519382308052	S100P	0.007923453771122
GPR89B; GPR89A; GPR89C	0.009685035752879	HNRNPAB	0.004556671182456	ITPRIP	0.008141793511831
TOMM70A	0.00985727829812	TGM2	0.004559272090698	CCT8	0.008179553129528
RPS3A	0.009912903092974	TUBGCP2	0.005365550535116	CSDE1	0.00826017763814
CPD	0.010358035005117	PPP2R1B	0.005367405307559	PPP1R7	0.00841905211178
FDFT1	0.011384097329045	MTHFD1	0.005656321008585	MYOF	0.008445362517772
S100A9	0.011424333483424	VCL	0.005964086861058	BSG	0.00849751844836
CSK	0.011457219717351	TLN1	0.006095596206959	TOR1AIP1	0.008581380621816
SCFD1	0.011479252278492	GOLPH3	0.006282031747958	CTPS1	0.008698647936996
SLC22A18	0.011486958799417	ANK3	0.006287454411422	BAIAP2	0.008954476729437
TCEB2	0.011536222884829	PSMB8	0.006422887698561	COPB1	0.009054941491304
HMGCS1	0.011566863040949	PDIA3	0.006503238027354	RALA; RALB	0.009125503281881
VAPA	0.011931203504948	CAP1	0.006525331944948	LOXL2	0.009333406602794
FLOT2	0.012423390656404	PSIP1	0.006552991186172	P4HA2	0.009570230984127
SEC22B	0.013264646154545	HNRNPH3	0.006637755565934	ATP5C1	0.009575490857271
RBM27; RBM26	0.013274920344656	MRPS23	0.00680490262868	ALDH18A1	0.009660205876263
CRKL	0.013329337406524	DLAT	0.006841330140428	BET1; DKFZp781C0425	0.009963704035784
PCK2	0.013422523676446	ACTB	0.006852554643281	CD46	0.009977977977685
EMC7	0.013446415975091	HTRA2	0.006917601786759	OPTN	0.010005503721279
GOLGB1	0.013539643669106	PTBP1	0.007037265329399	TWF1	0.010110476377996
OPA1	0.013620493118979	RRM2	0.007183363425031	SDPR	0.01023964305226
EPB41L2	0.013670914742496	ABCB6	0.007447140906832	METTL7A	0.010246137843823
CCDC47	0.013682725662798	EPPK1	0.007716005722363	PLXNB2	0.010297308727828
RANBP2	0.014264930587397	MBOAT7	0.008214656935344	ITPR3	0.010360522601888
TET1	0.014465089526579	IGF2BP3	0.008483815857937	DDX5	0.010396762419392
NOP56	0.014812651064485	ANXA4	0.008831706454686	LDHB	0.010525314892529
FAM3C	0.014949945967253	NAT10	0.008889749507977	DPF2	0.010718245540652
DHCR24	0.015258898631198	ZMYM6NB	0.009028705123634	PALLD	0.010862863685376
MMP1	0.015503619770998	DCTN1	0.009104149717639	ACSL1	0.011050727900649
VDAC2	0.015569953952931	RAB23	0.009171892429894	SLC39A8	0.011224351738872
ACSL1	0.016042813950581	NPEPPS	0.009338158807476	MRC2	0.011351052477394
MTDH	0.016467528303085	MYH9	0.009356838244682	CAPRIN1	0.011423927859134
S100A2	0.016741133495714	ITGB5	0.009621639112021	AHCYL1	0.011538336028154
DDX54	0.016898811285716	POLD3	0.009700671180402	UBXN1	0.011710709461661
HAT1	0.016957551894552	USP39	0.010080371666871	G3BP1	0.012190186940909
TNKS1BP1	0.017038488014705	NOL10	0.010199257943419	NCLN	0.012191757044491
MRPS5	0.017242150144882	PTPN11	0.010298708433261	GARS	0.01226993645158
ATP5F1	0.017482591955095	MTFP1	0.010508653017005	GLTSCR2	0.012373642409443
LRRC59	0.017497736466554	ACLY	0.010630225846536	H1F0	0.012391159774542
PAM16; CORO7	0.017505705670074	PA2G4	0.010711255263334	RAB5C	0.012451264397786
RPL13A	0.017799573413327	HNRNPL	0.010727648432897	EIF4E	0.012604842707167
CACYBP	0.018081655057736	UBA1	0.010749252116201	EDF1	0.012974829602012
MRPS7	0.018405887613552	SQRDL	0.011052780553527	HPCAL1; HPCA	0.013023264696894
HSPA8	0.018674160720574	SLC16A3	0.011057538177478	SLC4A7	0.013417930187314
GFPT1	0.019219175579789	ITGA2	0.011181808114291	MKI67IP	0.013640282288026
TMEM205	0.019228425633663	CPD	0.011315664438743	BAG3	0.013646748366157
ATAD3A	0.01937322947724	CHID1	0.011342362913486	IMPDH2	0.013713496902997
CLGN	0.019856100758262	HSPB1	0.01145525870626	ADAR	0.013722617067255
NOC3L	0.019910881368507	HNRNPA2B1	0.01147435299909	ASPH	0.014124565945207
KIAA1033	0.019996134058914	PITPNB	0.011493657017458	RANBP3	0.014132905197258
CFL1	0.020207610065077	WDR1	0.011563980610722	DCTN2	0.014218014455009
SLC25A11	0.020271584440865	NSF	0.011751090703484	MTHFD1L	0.014342746950884
SRPRB	0.020369401057665	SF3B14	0.011924177678341	ANXA11	0.014558574946331
PTCD3	0.020558894838134	MT-ATP8	0.012389248827531	FANCI	0.014562181909303
CPNE2	0.020585953677876	DNPH1	0.012636746006702	UCHL3	0.014827502676493
RFC3	0.020846587202663	UTP18	0.012738202273522	GCLC	0.014856681473226
PRDX4	0.021087968519987	MAT2B	0.012777067848653	PPAT	0.015390532359132
ABCE1	0.021206336900293	SRP9	0.013124370158829	TPM4	0.01541570246255
SF3A2	0.021216685239066	DNAJC9	0.013128026104643	CDC42BPB	0.016024236255626
SLC25A6	0.021233985596989	ZYX	0.013491194541454	BRE	0.016099983995371
PPP4R1	0.021237080466552	MKI67	0.013665798111442	ATXN2L	0.01620499824511
UBE2L3	0.021755378971892	GRHPR	0.013687394641254	SUN2	0.016415792225076
PLP2	0.022423464596259	AHCY	0.013864741954791	BAZ1B	0.01647568873437
B4GALT1	0.022497175851742	ACTN1	0.013869944769392	DHRS1	0.016925427778175
SART3	0.022539149889191	PKM	0.014033052062318	CCT5	0.017549165654663
FAF2	0.022613502238812	SDPR	0.014314176889041	FN1	0.017742774619541
RPL3	0.022884175151928	DTX3L	0.014813886286115	GMPPA	0.017827086566209
TPT1	0.023064459976129	EEF2	0.014913482699055	TPX2; HCA90	0.018467398710235
QPCTL	0.023530312264859	PFKP	0.014921308173293	UBR5	0.018585889733867
DCTN4	0.023533047019734	SERPINB3; SERPINB4	0.015142098664205	EEF1E1; hCG_2043275	0.018764134227863
GBP1	0.023860206485164	OPA1	0.015651013812695	SFXN3	0.018899390510519
ACAA2	0.024273116872023	FAM210A	0.016095938399802	TFAM	0.018907895372525
ITGA6	0.024460779682392	MCM7	0.01638235449348	COX5A	0.018990409820182
GNL3	0.024461585314268	PSMB5	0.01645743175702	LAS1L	0.018996290885632
FKBP10	0.024764899231212	MRPS17	0.016607562048242	SLC22A18	0.019020470664245
ATP5L	0.025053126305267	CISD1	0.016738285723702	PTGFRN	0.019205314758441
FUS	0.025295216312364	SMS	0.016881618933555	RAB9A	0.019233735516008
GSTK1	0.025297999125265	PABPC4	0.016959793878621	SPINT1	0.019306208101242
IDI1	0.025769120572354	RANBP2	0.01709520508628	LRRC47	0.019600642191467
LARP1	0.026090019191885	MRPL19	0.017191129809307	MPHOSPH10	0.019901101590421
GM2A	0.026211202662908	AKR1B1	0.017235118648035	POR	0.019943506452004
S100P	0.026242159423751	FLNB	0.017528246914698	GTPBP4	0.020411163453904
TIMM50	0.026368881718374	RAB8A	0.017657492031244	DYNC1LI2	0.020521673214148
EEF1A1; EEF1A1P5	0.026455458999469	MYBBP1A	0.017683339264619	TPM3; DKFZp686J1372	0.020673961516362
RPL6	0.026519050886935	TSG101	0.018032135874411	SLC16A3	0.021122829920431
COL12A1	0.027125327550966	ATP5B	0.018168971784517	SRSF9	0.021385364052327
CTTN	0.027189965784027	ANP32B	0.018351834807075	PLOD1	0.021420536109697
MIF	0.027373625049678	NRD1	0.018369336182959	ZNF596	0.021439577608275
LONP1	0.028054520312259	DTYMK	0.01839857971388	ELAVL1	0.021549645217667
TPD52	0.028902183670432	LRP1	0.018478130155762	CLDN1	0.022249291134437
APOBEC3B	0.029357203718889	LYAR	0.0188599510884	NYNRIN	0.022291201155762
PDLIM4	0.029371583486424	PDLIM5	0.01948578154129	SRRM2	0.02237088061985
CAPZB	0.02947534824956	CORO1C	0.020139145475688	DSC2	0.02241485891727
MYO1B	0.029690706412625	IARS	0.020508774163267	8-Sep	0.022572895644673
SACM1L	0.029695559920528	TMEM70	0.020511233657948	MUC1	0.022916287179215
MRPL17	0.029762529190204	SNRPA	0.020798326139302	SSR3	0.023303188090899
WASF2	0.030141500513569	EZR	0.02080090838173	GYS1	0.023356274345637
TBCA	0.030527010444042	GYS1	0.02093260272211	CPD	0.02364192803677
DHCR7	0.030543737241943	VDAC1	0.021055014454826	TAP1	0.023677781729719
KDSR	0.030672727525867	CSNK1A1	0.021118216399513	BRI3BP	0.024016437595199
MRPL42	0.0317278166702	HEATR1	0.021296660083121	CD81	0.024030848272941
NUP155	0.03181166305823	GTF3C5	0.021319312828767	MBOAT7	0.024086510679547
TGM2	0.031894083637145	SDHA	0.021608258885608	RPS18	0.024386137116713
ATP2A2	0.031908559073805	PTPRF	0.021792994509922	EIF4G2	0.024613866175271
TM9SF1	0.031969306896574	CSTF2; CSTF2T	0.022096066282373	PRKCDBP	0.024696481731597
MKI67	0.032040264783722	SSR1	0.022175969376362	LMNB2	0.024696723593748
RRM1	0.032088167495377	TOMM22	0.022358732666662	IFI16	0.024736716054353
DHRS3	0.032258811063461	APEH	0.022467677613292	ARIH2	0.025006913139186
OSGEP	0.03242892500434	HADHB	0.022681285577697	UGDH	0.02512329776908
RAB6A	0.032730965260824	BRD3	0.02279708211803	C2CD2	0.025135919635132
SLFN5	0.032732086192529	SET	0.022803164810375	CAMK2D	0.025249701165095
SNX9	0.032797473993557	MCM5	0.023085178488747	EIF3I	0.025324087949335
EIF2S2	0.033225974846872	ILVBL	0.023210248816811	VAV2	0.025366388837686
KIF2C	0.033384237238931	HSPG2	0.023743425872312	CYC1	0.025404790177633
TUFM	0.033811311367651	AHNAK	0.023822441798008	PKP2	0.025591616673106
GNB1	0.034028660351212	HNRNPM	0.02411217158064	EFHD2	0.025613990078106
SEC11C	0.034106823114781	SLC12A4	0.024272054796737	PES1	0.02572307386099
SLC4A7	0.034747621219689	ERAP1	0.024426303170543	EGFR	0.026908186291107
SRP9	0.035138860484259	HNRNPC; HNRNPCL1	0.024597310128795	DDAH1	0.027054115571908
PGD	0.035356250545387	VCP	0.024803095886508	ATP13A1	0.027314433301865
JUN	0.036228767313019	SNRPE	0.024813200343911	HNRNPUL2; hCG_2044799	0.027324595301086
DDX21	0.036700475064551	CBX3	0.024981524816091	GTF2E2	0.027406991781637
KRT18	0.036856025142515	CCT8	0.025115535177894	HK2	0.02748451561476
PCNA	0.03703869064914	ATIC	0.025223176492831	HSPA8	0.027985176196406
SNRPC	0.038110046554018	PSMD11	0.025267203503216	GIPC1	0.027990313755136
MRPL11	0.038492944430456	NOC2L	0.025507775121587	OPA1	0.028148787025882
NDUFV1	0.039024412147863	AGK	0.025882023915	COX6C	0.028260177789986
ALG5	0.039052078257846	MRPS22	0.025949589587507	PTRF	0.02896729832085
8-Sep	0.040059500866036	43164	0.026010316117037	VCL	0.029031421288978
UBXN4	0.0400967416522	HMOX2	0.026416445783298	HSPH1	0.029244507810198
ACADVL	0.040240933560833	SHCBP1	0.026583208170188	PCNA	0.029277631478876
ARHGEF2	0.040254560251934	SFRS3; SRSF3	0.027019821067031	EIF4A1	0.029479968919527
PPIA	0.040306648839516	HSPE1	0.027137205726697	CCT7	0.029759672121472
HNRNPA0	0.040659089090388	CCT7	0.02714854364587	RBMS2	0.029900191654786
H1FX	0.040660880674766	RNMT	0.027253996052488	TBL3	0.029995552557221
RSL1D1	0.040727388100261	SF3A1	0.027345766680684	COPB2	0.030143004032391
PCBP1	0.041060444256767	MPHOSPH10	0.027422953397422	CD36	0.030243794347784
OAT	0.041086025424954	FKBP8	0.027740329905471	ZNF207	0.031127518695247
RPS6	0.041548246869191	ANXA1	0.027806028691289	NAE1	0.03116262124055
CSRP1	0.041946757227102	STXBP3	0.027810703762034	CKAP4	0.031294490810304
IMPDH2	0.041995675064809	PAF1	0.028043692670092	AHCY	0.03165947398025
FASN	0.042330735683394	LSM14A	0.028431533445829	SDHC	0.03168187879169
HMGA1	0.042488060386211	GAPDH	0.028636042671187	TGOLN2	0.0320690029155
METAP1	0.042807060161675	DHRS7	0.028796767365608	SARS	0.032070856673193
C8orf33	0.043227193515104	LMNA	0.028871863087915	RAN	0.032198960301805
TPM1	0.043608051290617	UQCRB	0.029024329069001	IPO5	0.032255099460764
RAN	0.043765731081875	ERLEC1	0.029083387174762	PPP2R5E	0.032520779421543
CCDC22	0.043824377519062	SQSTM1	0.029169195075913	C16orf58	0.033359174417097
RAB10	0.044466049522161	COPA	0.029274244672455	EIF1; EIF1B	0.033507840309973
LARP4B	0.044951519016979	TMEM41B	0.029418154814256	MAOA	0.033991984285425
MYBBP1A	0.044959031659762	TMEM201	0.029507601823265	ITGA5	0.03430524174733
AGK	0.045256893668999	CYR61	0.029522502556828	SUN1; UNC84A	0.034560197490484
DDX6	0.045551723070911	UPP1	0.029535838877329	DDX21	0.034663482213619
C11orf48	0.046089436630983	UBA2	0.029565249998906	TMEM147	0.034725012172955
NSDHL	0.046589087554638	TSNAX; DISC1	0.029918634422305	CSRP2	0.035307269369821
NOP10	0.046752959712243	POP1	0.029922416844011	PKM; PKM2	0.036030516337652
RDH14	0.046770767991563	MFSD10	0.030916399699859	NMD3	0.03610741648908
SRP19	0.047035327924292	SLC25A6	0.030920334864811	OAS3	0.03622386344081
ARCN1	0.047037375951493	TMEM109	0.03097032505605	DSG2	0.036394241248742
TMED10	0.047087696669729	FAH; DKFZp686F13224	0.031115673654671	DPYD	0.036627222704875
SLC1A5	0.047114495205909	IVD	0.031137349382847	SRPK1	0.0371079748104
HN1L	0.047210340053632	AK2	0.031417238810662	ARPC3	0.037157040890348
DGAT1	0.047764365984012	WBP11	0.031459734874857	DAD1	0.037364429262822
DAD1	0.048049082283705	43358	0.032009608000601	METAP1	0.037466860624585
CDC37	0.048135690824494	EEF1E1; hCG_2043275	0.032323017024403	CIRBP	0.037578531107296
PDAP1	0.04822096561611	IDI1	0.032337941583088	P4HA1	0.037844551779456
ERGIC1	0.048327525975867	MT-ND4	0.032376042948936	BCAT2	0.038013706114236
RPL28	0.049026456822722	DCAF7	0.032852011030742	NRP1	0.038082753950453
IGF2BP2	0.049058985937291	ATP2A2	0.032936727886379	NDRG1	0.038373208406378
SFXN1	0.049195408876161	PDE12	0.033263239848297	UBE2L3	0.038591504225394
UNC45A	0.049615153151326	ACO1; IRP1	0.03328535637132	CD97	0.038860065477331
SCD	0.049680382150559	LETM1	0.033936598315055	TMEM2	0.038969995613597
		PHB2	0.03397175405248	PYGB	0.039081679189421
		ITGA1	0.034266242543753	TMSB10	0.039963848570791
		ZNF346	0.035407962723055	SEC22B	0.04049143360414
		NDRG1	0.035527696328217	MYL6	0.04049832981817
		ERP29	0.035576077357172	PHB	0.040619099392457
		C3	0.03580248449937	MGST3	0.040889045475386
		TCP1	0.035861077110922	STXBP2	0.04094875851427
		PVR	0.035894764662706	GPRC5A	0.040953982772269
		FASN	0.036002208550064	VPS4A	0.040955809249011
		CCT2	0.036332378659438	LDLR	0.04131058146994
		GSR	0.036419796652096	PDCD6IP	0.041603615762278
		HNRNPA0	0.03673880791215	PRMT5	0.041667495894254
		PSMD12	0.037431136201535	ALDH9A1	0.041752535855664
		NUSAP1	0.037826127496952	SMARCA4; SMARCA2	0.042011999483051
		CENPV	0.038581116557614	SMPD4	0.042295434605128
		NUCB2	0.038802623857286	API5	0.042307237242215
		ANXA3	0.038841172531667	CORO1B	0.042448810515789
		NOL6	0.038987589115343	NME1	0.042592236969446
		SLC25A4	0.03913970311486	PHGDH	0.042665900795564
		ANPEP	0.039479772494105	LIMA1	0.042752962711963
		ACOT7	0.039623921007478	KPNA1	0.042906494618499
		TFAM	0.039707734968897	CCT3	0.043664703493753
		RPLP0; RPLP0P6	0.040087400766381	PSME3	0.043770201664382
		NUP153	0.040111740483302	DARS; DKFZp781B11202	0.044048713395684
		AK3	0.040163982093579	FNDC3B	0.04409099304968
		RRS1	0.040527898253699	PDCD4	0.044255002794194
		MPDU1	0.040875557468697	KARS	0.04428763149879
		PPME1	0.040893092710894	YWHAB	0.044435424781182
		NXF1	0.041071516841707	TUBA4A	0.044822390243966
		COLGALT1	0.041637645286223	HSP90AB2P	0.045275665873445
		TCERG1	0.041817433270138	MFF	0.045305132154466
		MYH14	0.04200459437687	SRI	0.045339113744608
		DKC1	0.042045226104732	SUMO2; SUMO3; SUMO4	0.045389142630342
		CHP1	0.042321931446982	SMU1	0.045693951967544
		ACADVL	0.04251795230916	PGRMC1	0.045854689656102
		YTHDF2	0.042557149834127	UFL1	0.045994516676888
		ITM2C	0.042664998944362	TMPO	0.046071527099456
		FNDC3A	0.043020485898521	GLG1	0.046210152745552
		DDX23	0.043041446364437	STX12	0.046233460411152
		TM9SF1	0.044250928692254	QARS	0.046266638866445
		RBM34	0.044452556043337	NDUFB8	0.046327835309821
		PARP1	0.044642062465197	NAGK	0.046635248106221
		HSPA5	0.044940359567747	UBA5	0.047018290706918
		LRRC8A	0.045128672116194	DCBLD1	0.047123009546817
		GNAI3	0.045215904061064	RMDN3	0.047126494670995
		APMAP	0.045332288978615	C3orf20	0.047396813435199
		ERVMER34-1	0.045751082576868	CPT1A	0.047454336018546
		SLC25A3	0.046011301133572	LRRC8A	0.047708173691333
		PSMD3	0.046034878028335	TNC	0.047757621338458
		LOC728763	0.0461135520587	PSMB7	0.047922090360813
		ACTR3	0.046140724299598	SLC25A11	0.048267993682209
		ABHD10	0.04614997610942	ITGB5	0.048350367510199
		DOCK11	0.046269853302418	RPA2	0.048873156537706
		PIN4	0.04659637792031	GBP1	0.049176725246684
		AHNAK2	0.046681926091011	PAPSS1	0.049526079782675
		NOC4L	0.0467975144642	NDUFAF2	0.049665450756475
		NACAP1	0.047591433048054		
		CD97	0.047882227230363		
		MT-CO3	0.047965144307878		
		SDHB	0.048181890662094		
		SMPD4	0.048403063887703		
		FIS1	0.048873636426099		
		IDH2	0.048977469868803		
		RPL26; KRBA2	0.049123691061664		
		ARPC1A	0.049507518430601		
		MPZL1	0.049733549424119		
		APP	0.049980781519213

**Table 4:** Fold Regulation of AgNP-treated epithelial cells compared to controls.

Gene ID	Chronic Treatment			Acute Treatment		
	Colon	Lung	Mammary	Colon	Lung	Mammary
ACTN1	−11.58	−1.45	1.02	−1.32	−1.20	−2.03
ACTN2	−108.44	1.13	−2.21	3.79	1.68	2.40
ACTN4	5.60	−1.14	−1.91	−1.21	−1.33	−1.08
ADAMTS1	−1.19	4.52	−1.06	1.88	1.12	2.05
ADAMTS13	−23.12	1.98	−2.70	−2.63	1.10	1.25
ADAMTS8	3.26	5.19	1.45	−1.60	1.21	1.07
AKT1	3.45	1.03	−1.77	−2.26	−1.35	−2.41
AKT2	1.83	−1.17	−1.45	−1.57	1.22	−10.50
AKT3	4.07	−1.29	1.09	−1.66	−1.42	−2.42
ANOS1	−9.45	2.74	−1.09	−3.79	−1.02	−1.04
ARHGAP5	1.67	−1.85	−1.10	1.16	1.87	−2.40
BCAR1	1.95	1.16	−2.11	−4.31	−2.58	−3.37
CAPN2	2.25	−1.44	1.02	−1.01	−4.95	−1.27
CAV1	70.30	−1.89	−1.05	−2.01	21.80	−1.18
CAV2	1.29	−1.41	−1.14	1.04	−2.96	−2.32
CAV3	−2.42	2.01	−1.10	−1.38	−1.79	4.91
CD44	−1.01	3.24	1.52	−1.63	−2.00	−1.37
CDC42	2.95	−1.43	−1.18	−1.03	−1.33	1.12
CDH1	2.10	2.49	1.68	2.28	1.05	−1.21
CLEC3B	1.43	2.11	4.44	−1.97	1.14	−1.09
CNTN1	4.55	1.04	10.76	−4.45	4.16	5.04
COL11A1	2.74	1.29	−1.09	−2.43	2.48	−8.89
COL12A1	1.42	1.77	1.94	−2.55	−1.70	−1.93
COL14A1	−1.08	9.75	4.90	−2.05	3.29	1.55
COL15A1	1.98	4.24	4.13	−1.13	−1.05	50.15
COL16A1	1.18	1.70	1.70	−1.37	−1.26	−1.21
COL1A1	−1.22	2.02	1.45	−2.30	−1.35	1.10
COL4A2	−1.20	2.31	2.50	−1.36	−1.29	−1.16
COL5A1	−1.87	−1.02	−1.09	−2.18	−1.61	−1.03
COL6A1	−1.87	2.24	−1.05	−1.20	1.16	−1.07
COL6A2	−1.22	1.78	−1.09	−1.72	−1.12	1.16
COL7A1	72.59	−1.28	−2.11	−2.39	1.74	1.06
COL8A1	1.15	−2.76	−1.38	−3.00	−1.67	−2.07
CRK	−65.28	−1.46	−1.42	122.21	2.41	−305.50
CRKL	−1.78	−1.51	−1.98	−1.36	1.28	−1.69
CTGF	1.58	3.02	−1.25	3.12	2.50	2.21
CTNNA1	−1.26	3.04	1.30	−1.60	−1.81	−1.28
CTNNB1	2.62	2.47	2.12	−1.34	−1.95	−1.29
CTNND1	−1.08	2.61	1.20	−1.76	1.21	−1.06
CTNND2	−177.49	1.15	−4.66	1.61	1.05	1.27
DIAPH1	−1.15	1.02	−2.04	−1.30	−1.57	−2.17
DOCK1	−132.75	−1.08	1.13	−2.40	1.12	−310.80
DST	3.25	1.24	−1.10	1.05	−2.66	−1.14
ECM1	−1.19	4.30	−1.41	−1.13	1.41	1.78
FLNA	1.14	1.20	−3.47	−1.12	−2.18	−1.04
FLNB	6.09	1.16	−1.45	1.23	−2.35	−1.25
FN1	1.01	1.78	1.69	−2.10	−1.71	−1.47
FYN	−1.50	−1.82	1.23	−1.47	1.16	1.15
GRB2	6.07	1.25	−1.64	−1.67	−1.48	−2.40
GSK3B	−57.24	−1.33	−1.04	2.18	1.51	−85.26
HAS1	1.32	29.56	−1.29	−2.81	14.56	1.01
HRAS	3.51	−1.79	−1.01	1.19	−29.33	−4.56
ICAM1	1.66	2.20	6.39	1.06	2.87	−2.05
ILK	−1.54	−1.99	−1.32	−2.19	−3.33	−3.54
ITGA1	2.14	2.37	2.66	−1.11	−1.38	−2.42
ITGA11	−2.44	−4.21	−1.04	−3.21	−2.68	−1.18
ITGA2	9.29	3.09	1.54	−1.48	−3.89	−1.90
ITGA2B	10.53	1.16	−1.05	−2.40	1.51	−1.18
ITGA3	1.15	1.05	−1.76	−2.26	−5.71	−3.16
ITGA4	11.88	−2.06	−1.52	4.55	1.03	−5.76
ITGA5	−2.20	1.99	1.86	−2.13	−1.93	−1.43
ITGA6	1.01	3.91	1.96	6.34	2.16	−1.39
ITGA7	−2.06	1.70	−1.74	−1.74	−1.64	−1.75
ITGA8	−1.88	4.58	−1.04	−2.40	−5.41	17.30
ITGA9	1.04	−2.50	−1.13	1.11	13.34	−2.04
ITGAL	−1.38	49.48	3.89	1.05	2.31	3.03
ITGAM	1.48	41.71	−1.87	4.91	10.54	−1.49
ITGAV	1.51	−1.94	1.90	−1.17	−1.90	−2.52
ITGAX	−1.43	1.50	2.94	−1.71	2.48	−2.24
ITGB1	4.44	3.31	1.67	−1.59	−2.04	−1.87
ITGB2	458.66	13.51	1.68	−1.23	2.74	−1.15
ITGB3	−429.33	3.19	3.86	3.41	−1.35	−751.03
ITGB4	2.35	1.52	−2.13	−1.36	−1.96	−4.64
ITGB5	65.35	2.29	1.54	−1.34	−2.43	−1.81
ITGB6	2.09	1.33	−1.22	57.41	1.51	89.06
LAMA1	−1.21	3.57	1.08	−1.72	1.73	−3.31
LAMA2	−5.12	2.78	1.06	1.30	1.58	3.47
LAMA3	−1.12	4.16	1.98	−1.96	−1.76	−2.12
LAMB1	1.25	3.17	1.45	−1.88	−2.01	−2.61
LAMB3	−1.07	4.61	1.67	1.65	1.13	−1.06
LAMC1	1.43	3.47	1.61	−1.74	−2.55	−1.61
MMP1	119.83	5.52	−1.68	1.03	−15.07	−3.54
MMP10	332.77	2.15	−1.06	5.00	−1.58	−1.02
MMP11	−1.76	1.20	−1.11	−5.23	−1.43	−1.57
MMP12	9.02	−1.14	−1.09	3.02	−5.43	−1.15
MMP13	42.09	2.99	−9.89	1.90	22.68	2.75
MMP14	−190.30	−1.08	−1.14	−1.44	1.12	1.10
MMP15	−3.25	−1.62	−1.39	−2.25	−1.08	−1.92
MMP16	1.07	3.15	−1.75	−2.09	−2.50	−1.77
MMP2	−5.23	1.59	1.07	−1.86	−1.32	1.41
MMP3	−1.03	2.42	−1.09	1.77	−25.52	−2.25
MMP7	−10.75	2.92	2.62	5.30	−1.49	1.21
MMP8	−1.19	62.20	−2.02	−2.74	5.32	−1.27
MMP9	−1.59	1.52	−1.01	1.13	1.65	3.22
NCAM1	1.07	1.85	1.90	−1.74	2.63	−2.03
PAK1	−86.56	−1.37	−1.04	−1.21	2.11	−1.85
PAK2	2.22	−1.58	−1.02	−1.17	1.26	−3.55
PAK3	−1.45	−1.30	−1.91	−1.96	−1.13	1.32
PAK4	−3.07	−1.14	−1.88	−1.28	−1.38	−2.18
PARVA	−160.35	−1.78	−1.22	21.04	−3.66	−4.55
PARVB	−171.77	1.14	−1.07	−1.24	−1.82	−2.74
PARVG	2.98	−1.08	1.09	3.30	1.75	1.36
PDPK1	−1.02	−1.36	1.07	−1.18	−1.08	−1.16
PECAM1	263.85	25.03	1.53	−1.30	3.40	−1.05
PIP5K1C	−647.33	1.41	−2.27	1.33	−1.40	−9.54
PLEC	1.44	1.50	−1.63	−11.15	−166.75	−288.35
PRKCA	−1.10	−1.22	1.08	−2.82	−3.33	−6.49
PRKCB	1.29	1.17	−1.31	5.58	−23.00	−15.76
PRKCG	−7.49	2.46	1.82	1.19	−1.28	384.04
PTEN	−1.20	−1.33	1.02	−3.95	−1.69	2.28
PTK2	−282.25	−1.36	1.05	−277.83	−9.17	−3.65
PXN	−2.26	−1.13	−1.56	−2.81	−6.39	1.73
RAC1	−1.47	−1.44	1.13	−1.76	−1.82	−3.90
RAC2	−229.22	−1.14	−1.59	−2.40	1.51	−1.18
RAF1	−561.97	−1.82	−1.12	−1.62	1.04	−7.03
RAP1A	2.23	−1.33	1.08	−1.34	−1.66	2.48
RAP1B	1.97	−1.14	1.22	−1.63	1.00	3.03
RAPGEF1	2.79	−1.16	−1.71	5.87	−2.75	−3.22
RHOA	2.31	−1.39	1.15	−1.85	1.24	2.38
ROCK1	−151.76	−1.53	−1.14	−17.80	−11.80	−2.02
ROCK2	−210.17	−1.64	1.07	−2.40	1.51	−1.18
SELE	10.23	2.56	−1.09	3.61	2.38	−1.36
SELL	−1.41	19.82	1.66	1.55	10.56	1.75
SELP	−1.22	1.04	−3.04	−1.17	7.81	−1.04
SGCE	1.11	2.45	1.43	−1.75	−2.14	−2.17
SHC1	2.60	−1.15	−1.68	788.14	−590.58	−1.18
SOS1	1.29	−1.18	1.04	−2.40	−608.22	−1.18
SOS2	−168.03	−1.44	−1.08	947.78	−4.02	−1.18
SPARC	−2.05	2.08	1.06	−1.71	−1.39	−1.01
SPG7	−2.29	1.47	−1.18	−2.77	−1.80	−1.62
SPP1	148.79	2.52	−1.19	−1.30	−273.37	1.05
SRC	1.38	1.18	−1.74	−1.97	1.47	−3.91
TGFBI	1.33	3.47	1.70	−1.44	−2.78	−1.43
THBS1	1.44	2.04	1.80	−2.20	−4.67	1.03
THBS2	187.76	1.71	1.90	−5.42	−11.97	1.32
THBS3	−3.06	1.91	−1.41	−3.02	−1.54	−1.27
TIMP1	−1.81	3.13	1.36	−2.11	−1.58	−1.10
TIMP2	−3.27	2.82	2.04	−1.83	−1.55	−1.12
TIMP3	−1.11	3.72	1.40	1.13	3.53	−4.32
TLN1	1.45	−1.35	−1.25	−2.40	1.51	−1.18
TNC	123.59	1.71	−1.08	−2.18	−2.29	1.80
TNS1	7.94	−1.09	−4.31	−1.58	−2.27	2.44
VASP	−1.62	1.07	−2.01	23.43	1.19	24.06
VAV1	−42.27	−1.29	1.97	−2.40	1.51	−1.18
VAV2	−1.13	1.06	−1.50	1.53	−3.15	1.63
VCAM1	1.01	1.05	−1.09	−2.30	−7.85	−1.04
VCAN	2.09	1.48	1.64	−2.32	−1.20	−1.17
VCL	−1.40	−1.35	−1.34	1.05	2.62	−2.94
VTN	−1.84	2.04	−1.81	−1.90	−1.71	−2.24
ZYX	−989.39	−1.71	−1.18	−1.42	−3.04	1.51

**Table 5:** IPA analysis derived from LC-MS/MS protein data of AgNP-treated colon cells IPA Summary: Colon.

acute	chronic	change
**Top Canonical Pathways**		
Colanic Acid Building Blocks Biosynthesis	RhoA Signaling	Superpathway of Cholesterol Biosynthesis
Glycolysis I	Remodeling of EpithelialAdherens Junctions	Cholesterol Biosynthesis I
NER Pathway	Hypoxia Signaling in theCardiovascular System	Cholesterol Biosynthesis II (via 24,25-dihydrolanosterol)
UDP-N-acetyl-d-galactosamine Biosynthesis II	Epithelial AdherensJunction Signaling	Cholesterol Biosynthesis III (via Desmosterol)
Cardiac -adrenergic Signaling	Actin Cytoskeleton Signaling	Mevalonate Pathway I
**Top Upstream Regulators**		
let-7	XBP1	SREBF2 (inhibited)
mir-122	COLQ	SCAP (inhibited)
CNTF	SORT1	ERBB2
HCAR1	mir-122	INSR
PDLIM1	PGR	POR (activated)
**Fold Change Up**		
APOB (+36.7X)	SUMO1 (+4.9X)	MFN2 (+5.8X)
ACTBL2 (+13.4X)	PPIE (+4.6X)	RDH14 (+4.7X)
ARFGEF1 (+10.7X)	TET1 (+3.3X)	MMP1 (+4.3X)
PPT1 (+5.2X)	RPP30 (+3.1X)	CPD (+3.7X)
TM9SF1 (+4.3X)	ARFGAP3 (+3.0X)	S100A9 (+3.2X)
CSK (+3.6X)	PHPT1 (+3.0X)	TET1 (+3.0X)
MRPL42 (+3.3X)	BBOX1 (+2.8X)	MRPL22 (+3.0X)
PHPT1 (+3.0X)	UNC45A (+2.6X)	DHRS3 (+2.8X)
PPIE (+2.9X)	AGRN (+2.3X)	MRPS5 (+2.7X)
SPG7 (+2.8X)	GOLGB1 (+2.1X)	S100P (+2.6X)
**Fold Change Down**		
ITGA7 (−14.9X)	HMGN1 (−11.7X)	HMGCS1 (−62.5X)
EPPK1 (−13.7X)	GPD2 (−9.1X)	MKI67 (−21.0X)
SPC25 (−6.9X)	GPX1 (−8.9X)	FDFT1 (−16.5X)
MFN2 (−5.2X)	GSTK1 (−6.3X)	COL12A1 (−6.7X)
HYDIN (−3.9X)	IER3IP1 (−5.8X)	S100A2 (−6.5X)
TCP11L1 (−3.9X)	MSH3 (−4.9X)	DHCR24 (−6.4X)
TMEM189 (−3.6X)	EPB41L2 (−4.2X)	LDLR (−5.5X)
TET1 (−3.3X)	VAMP7 (−3.9X)	MSMO1 (−5.4X)
PPL (−3.2X)	ATP6AP1 (−3.2X)	GSTK1 (−5.2X)
RABGGTA (−3.2X)	DUT (−3.0X)	VAMP3 (−5.2X)
**Molecular and Cellular Functions**		
Cellular Assembly and Organization	Drug Metabolism	Protein Synthesis
Cellular Function and Maintenance	Molecular Transport	Lipid Metabolism
RNA Post-Transcriptional Modification	Cellular Function and Maintenance	Small Molecule Biochemistry
Cellular Movement	Cell Morphology	Vitamin and Mineral Metabolism
Carbohydrate Metabolism	Cellular Assembly andOrganization	Molecular Transport
**Top Tox Lists**		
Cardiac Necrosis/Cell Death	Hypoxia-Inducible Factor Signaling	Cholesterol Biosynthesis
NRF2-mediated Oxidative Stress Response	Mitochondrial Dysfunction	Oxidative Stress
LXR/RXR Activation	Oxidative Stress	Cardiac Necrosis/Cell Death
Positive Acute Phase Response Proteins	Increases Renal Proliferation	Fatty Acid Metabolism
Increases Liver Damage	Increases Depolarization of Mitochondria and Mitochondrial Membrane	Cardiac Hypertrophy

**Table 6:** IPA analysis derived from LC-MS/MS protein data of AgNP-treated Lung cells.

acute	chronic	change
**Top Canonical Pathways**		
EIF2 Signaling	EIF2 Signaling	Remodeling of EpithelialAdherens Junctions
Sirtuin Signaling Pathway	TCA Cycle II (Eukaryotic)	Integrin Signaling
TCA Cycle II (Eukaryotic)	NRF2-mediated Oxidative Stress Response	Epithelial AdherensJunction Signaling
Telomere Extensionby Telomerase	Glycolysis I	Sirtuin Signaling Pathway
RAN Signaling	Gluconeogenesis I	Mitochondrial Dysfunction
**Top Upstream Regulators**		
TP53	TCR	TP53 (activated)
MYC	TP53 (inhibited)	MYC
LONP1	MAPT	MMP12
CST5 (activated)	PSEN1	EGFR
PCGEM1	APP	LONP1
**Fold Change Up**		
CSTF3 (+9.8X)	MX2 (+19.4X)	ACSL1 (+28.6X)
MAL2 (+3.8X)	IFIT3 (+11.5X)	CA9 (+27.5X)
NME1 (+3.1X)	OASL (+5.1X)	YTHDF2 (+10.8X)
VPS4A (+3.0X)	CLPB (+4.5X)	TGM2 (+10.1X)
SNW1 (+2.9X)	MRPL2 (+4.2X)	MECP2 (+9.4X)
DLST (+2.8X)	COX20 (+3.9X)	DOCK11 (+8.5X)
NDUFA7 (+2.8X)	ITGA2 (+3.9X)	HEATR1 (+7.9X)
GRHPR (+2.8X)	SLK (+3.6X)	MT-ND4 (+7.9X)
SRSF11 (+2.6X)	RRS1 (+3.6X)	NDRG1 (+7.6X)
NUSAP1 (+2.5X)	UTP4 (+3.3X)	PTPRF (+7.6X)
**Fold Change Down**		
SLC38A2 (−18.3X)	MACROD1 (−8.4X)	SQSTM1 (−14.9X)
MT-ND4 (−8.5X)	BCAT2 (−6.9X)	CYR61 (−3.6X)
MRPS17 (−8.3X)	DDAH2 (−6.5X)	SERPINB4 (−3.1X)
F3 (−8.3X)	PPT1 (−6.0X)	SERPINB3 (−3.1X)
MRPL40 (−7.3X)	NIT2 (−5.4X)	IDI1 (−3.0X)
SLC12A4 (−6.7X)	TBRG4 (−4.4X)	NUSAP1 (−2.9X)
ITPR3 (−6.2X)	PTGR1 (−3.7X)	NRDC (−2.9X)
TGM2 (−5.7X)	WASL (−3.5X)	APEH (−2.9X)
MT-ATP8 (−5.7X)	COL12A1 (−3.4X)	GRHPR (−2.9X)
UTP18 (−5.2X)	PEA15 (−3.1X)	FNDC3A (−2.8X)
**Molecular and Cellular Functions**		
RNA Post-Transcriptional Modification	RNA Damage and Repair	RNA Post-TranscriptionalModification
Protein Synthesis	Protein Synthesis	Cell Death and Survival
RNA Damage and Repair	Cell Death and Survival	Cellular Development
Cell Death and Survival	RNA Post-Transcriptional Modification	Cellular Growth andProliferation
Molecular Transport	Free Radical Scavenging	Protein Synthesis
**Top Tox Lists**		
Mitochondrial Dysfunction	NRF2-mediatedOxidative Stress Response	Mitochondrial Dysfunction
TR/RXR Activation	Renal Necrosis/Cell Death	Cardiac Necrosis/Cell Death
Renal Necrosis/Cell Death	Aryl HydrocarbonReceptor Signaling	NRF2-mediated OxidativeStress Response
Cardiac Necrosis/Cell Death	Oxidative Stress	Decreases TransmembranePotential of Mitochondria and Mitochondrial Membrane
Hypoxia-Inducible Factor Signaling	Fatty Acid Metabolism	Decreases Depolarization ofMitochondria and Mitochondrial Membrane

**Table 7:** IPA analysis derived from LC-MS/MS protein data of AgNP-treated mammary epithelia IPA Summary: Breast.

acute	chronic	change
**Top Canonical Pathways**		
EIF2 Signaling	Sirtuin Signaling Pathway	Regulation of eIF4 andp70S6K Signaling
Sirtuin Signaling Pathway	Heme Degradation	Methionine DegradationI (to Homocysteine)
Mitochondrial Dysfunction	TCA Cycle II (Eukaryotic)	Cysteine BiosynthesisIII (mammals)
Remodeling of EpithelialAdherens Junctions	Mitochondrial Dysfunction	Caveolar-mediatedEndocytosis Signaling
Oxidative Phosphorylation	Actin Nucleation byARP-WASP Complex	PI3K/AKT Signaling
**Top Upstream Regulators**		
TP53	KDM5A	TP53
MMP12	Esrra	HSF1
HSF1	miR-149-5p	PGR
CST5 (activated)	miR-291a-3p	HIF1A
RICTOR	Collagen type III	IL5
**Fold Change Up**		
CRNKL1 (+14.6X)	HMOX1 (+12.4X)	PROM2 (+19.9X)
AASDHPPT (+5.1X)	CWF19L1 (4.7X)	PTPRF (+19.1X)
MYADM (+5.0X)	SEC14L2 (+4.3X)	LRP1 (+17.9X)
NCAPH (+4.6X)	PPP1R7 (+3.2X)	HMOX1 (+12.4X)
OPTN (+4.3X)	SPRYD4 (+3.1X)	FN1 (+12.3X)
GFM1 (+3.5X)	DDX27 (2.9X)	CA12 (+9.2X)
GTPBP1 (+3.5X)	ARPC5 (+2.9X)	ZNF596 (+8.6X)
TRIOBP (+3.4X)	FIP1L1 (+2.6X)	PRSS53(+6.4X)
TMPO (+3.0X)	RPS19BP1 (+2.5X)	VKORC1 (+6.4X)
RILPL1 (+2.9+)	CTBP2 (+2.2X)	PDCD4 (+6.3X)
**Fold Change Down**		
NIP7 (−9.0X)	HYDIN (−7.6X)	CRNKL1 (−12.1X)
PLXNA2 (−4.8X)	ATP6AP2 (−6.3X)	TNC (−9.6X)
PROM2 (−4.5X)	SWAP70 (−4.4X)	NOP53 (−8.9X)
IFITM3 (−3.6X)	GEMIN4 (−3.8X)	ATP6AP2 (−8.5X)
IFITM2 (−3.6X)	ARID1B (−2.8X)	CRTAP (−7.9X)
PTGS1 (−3.4X)	RNMT (−2.8X)	TMSB10/TMSB4X (−5.7X)
NUCB2 -(3.2X)	BAZ1B (−2.6X)	RBM3 (−5.2X)
PTPRF (−3.2X)	NUDCD2 (−2.5X)	NYNRIN (−5.2X)
CDC42BPB (−3.1X)	TRIP13 (−2.3X)	PALLD (−4.8X)
TRIM56 (−2.9X)	HPF1 (−2.2X)	NRP1 (−4.8X)
**Molecular and Cellular Functions**		
Cell Death and Survival	Protein Synthesis	Cell Death and Survival
RNA Post-Transcriptional Modification	Cell Morphology	Protein Synthesis
Protein Synthesis	Cellular Function andMaintenance	Protein Degradation
Post-Translational Modification	Cell Death and Survival	Cellular Development
Protein Folding	Cell-To-Cell Signaling andInteraction	Cellular Growth andProliferation
**Top Tox Lists**		
Mitochondrial Dysfunction	Mitochondrial Dysfunction	Mitochondrial Dysfunction
NRF2-mediatedOxidative Stress Resp.	Long-term Renal InjuryAnti-oxidative Resp. (Rat)	Xenobiotic MetabolismSignaling
Cell Cycle: G2/M DNA Damg. Chkpt. Reg.	Positive Acute PhaseResponse Proteins	Hypoxia-InducibleFactor Signaling
Cell Cycle: G1/S Checkpoint Regulation	Protect. fr. Hypox.-inducedRenal Ischemic Inj. (Rat)	Cardiac Necrosis/Cell Death
Renal Necrosis/Cell Death	Incr. Transmb. Pot'l. of Mitoand Mito Mb	NRF2-mediatedOxidative Stress Response

Supplementary data includes Table S1 (consisting of raw MS LFQ intensity values used to generate Table 1, Table 2, Table 3), and PDF files of IPA summaries of LC-MS/MS data from all three cell lines at all three AgNP exposure conditions (acute, chronic and changes over time (OT)), which were used to generate Table 5, Table 6, Table 7

## 2 Experimental design, materials and methods

### 2.1 Cell Culture

MCF10A, BEAS2B, and CCD-18Co cell models were obtained from American Type Culture Collection (Manassas, VA). Cells were cultured as directed by the supplier and all epithelial cells were cultured in “standard growth medium” (DMEM containing 10% FBS) during AgNP exposure for consistency.

### 2.2 AgNP Exposure

Each cell line was plated at concentrations predetermined to establish a confluent cell monolayer within three days. Cells were then cell cycle synchronized overnight in serum-starved medium, and then treated with 40 nm AgNP or AgNP diluent in standard growth medium for 6 or 24 hr. For chronic AgNP treatment, serum-starved confluent cells were treated for 24 hr with 40 nm AgNP or diluent (control cells) and then replaced with fresh medium (-AgNP) and cultured for eight days, with changes in medium every 2–3 days as needed.

### 2.3 Filter-aided Protein Sample Preparation

Cell pellets were subjected to filter-aided sample preparation [4] for protein cleanup using Vivacon 30,000 kDa molecular weight cutoff filters and reconstituted with 100 μL of 50 mM ammonium bicarbonate solution. Tryptic digestion was carried out by hydrating lyophilized trypsin to a stock solution of 1.0 μg/μL with 0.01% acetic acid in water; trypsin was added to the sample mixture at a 1:50 (v/v) ratio and then incubated at 37 °C for 4 hr. After digestion, peptides were acidified with HCl to a final concentration of 250 mM (pH ≤ 3) and aliquoted for LC-MS.

### 2.4 Nanoflow Liquid Chromatography (LC)

Pico-frit columns were purchased from New Objective (Woburn, MA) and packed to a length of 20–30 cm with reverse phase ReproSil-Pur 120 C-18- AQ 3.0 μm particles (Dr. Maisch GmbH HPLC, Ammerbuch-Entringen, Germany). Peptide separation was achieved on the column by injecting 2.0 μL of sample and using a gradient of mobile phase A (98.0% water, 2.0% acetonitrile, and 0.1% formic acid) and mobile phase B (80.0% acetonitrile, 20.0% water 0.1% formic acid). The LC method consists of a 120 min gradient with a linear ramp from 0.0% B to 40.0% B, a 1 min ramp to 100% B which is held for 6 min (123–129 minutes), followed by equilibration of the column at 0.0% B (130–140 minutes) running at a constant flow rate of 300 nL/min.

### 2.5 Orbitrap Mass Spectrometry (MS)

Orbitrap tandem mass spectrometry was performed using a Thermo Scientific Q-Exactive HF (Bremen, Germany) in a top 20 data dependent acquisition mode (DDA), where the 20 most abundant precursors were selected for fragmentation per full scan. MS1 and MS2 scans were performed at a resolving power of 120,000 and 15,000 at *m*/*z* 200, respectively. A dynamic exclusion window of 20 seconds was used to avoid repeated interrogation of abundant species. Automatic gain control was 1e6 and 1e5 for MS1 and MS2 scans, respectively. Samples were run in random order, and a quality control BSA digest was run and monitored every fifth injection to ensure proper LC-MS/MS reproducibility using AutoQC [5].

### 2.6 Protein Identification

Resulting raw data was loaded into MaxQuant (Version 1.5.6.0) [6], wherein MS/MS spectra were searched against a human proteome FASTA file downloaded from the Swiss-Prot protein database. The search included variable modifications of methionine oxidation and N-terminal acetylation, and fixed modification of cysteine carbamidomethylation. Peptides of a minimum of eight amino-acids and a maximum of two missed cleavages were allowed for the analysis. The peptide and protein identification false discovery rate (FDR) was set to 0.01. The resulting proteinGroups.txt data was imported into Perseus (Version 1.6.2.1) [7]. Here, reverse proteins or those only identified by site were filtered out. Next, the LFQ Intensity data was log 2 transformed and those proteins that did not produce a valid value in a minimum of two out of the three replicates in at least one group (i.e., proteins that were only detected once within a triplicate group), were filtered out. All remaining missing values were imputed using a normal distribution with a width of 0.3 and downshifted 1.8 standard deviations. Contaminants were then removed and group comparisons were performed using a two-sample student's t-test utilizing a Benjamini-Hochberg FDR calculation set to 0.05 for truncation. Table 1, Table 2 summarize changes in protein expression induced by acute and chronic AgNP treatment, respectively, of BEAS2B (lung), MCF10AI (breast), and CCD-18Co (colon) cultured epithelia as determined by LC-MS/MS. Table 3 summarizes changes in protein expression between 6 hours and 8 days in the three epithelial cell lines.

### 2.7 Quantitative Real Time PCR Arrays

Total RNA was isolated using the RNeasy kit (Qiagen; Hilden, Germany). RNA was reverse transcribed using the RT^2^ First Strand Kit, and RT^2^ Profiler PCR arrays (for focal adhesions, ECM and adhesion molecules) were used according to the manufacturer's instructions. Relative fold changes in gene expression were determined via the ^ΔΔ^CT method using online analysis tools provided by Qiagen. Genes altered by acute and chronic AgNP exposure in BEAS2B (lung), MCF10AI (breast), and CCD-18Co (colon) cultured epithelia are found in Table 4.

### 2.8 Ingenuity pathway analysis (IPA)

Protein and mRNA datasets were imported into IPA for functional analysis (Qiagen, https://www.qiagenbioinformatics.com/products/ingenuity-pathway-analysis). The most significant networks and canonical pathways were predicted in IPA using restrictive statistical parameters to identify pathways affected by significantly altered proteins or mRNAs. Algorithms defining networks and pathways are drawn from the Ingenuity Knowledge Base, a large, manually curated collection of nearly 5 million findings from the biomedical literature or integrated from third-party databases [8]. Canonical pathways classify molecules in the given dataset as per their reported ultimate biological function. Pathway significance is indicated by the number of molecules represented in the provided dataset with respect to the total number of identified molecules reported to affect the specific biological function. In the representative figures, calculated z-scores indicate top canonical pathways based on altered protein levels for the three epithelial cell lines exposed to acute (Fig. 1) and chronic (Fig. 3) AgNP exposure, as well as analysis of changes in protein expression between acute and chronic levels (Fig. 5). The ratio (orange dots connected by a line) indicates the ratio of proteins from the dataset that map to the pathway divided by the total number of genes that map to the same pathway. Primary causal network analysis of acute (Fig. 2) and chronic (Fig. 4) AgNP exposure and analysis of changes in protein expression between acute and chronic levels (Fig. 6) draws from approximately 40,000 nodes that represent mammalian genes and their products, chemical compounds, microRNA molecules and biological functions. Nodes are connected by approximately 1,480,000 edges representing experimentally observed cause–effect relationships that relate to expression, transcription, activation, molecular modification and transport as well as binding events. Top canonical pathways and primary causal networks were also determined from QPCR pathway directed microarray data (Fig. 7).
